# Vericiguat protects against cardiac damage in a pig model of ischemia/reperfusion

**DOI:** 10.1371/journal.pone.0295566

**Published:** 2023-12-22

**Authors:** Weida Zhu, Yue Ben, Yang Shen, Wenbing Liu

**Affiliations:** 1 The Department of Cardiology, Yizheng Hospital of Nanjing Drum Tower Hospital Group, Yizheng, Jiangsu, China; 2 The Department of Cardiology, Affiliated Hospital of Nantong University, Nantong, Jiangsu, China; 3 Department of Cardiovascular Medicine, Affiliated Maternity and Child Health Care Hospital of Nantong University, Nantong, Jiangsu, China; Tanta University Faculty of Medicine, EGYPT

## Abstract

**Background:**

The purpose of this research was to verify that vericiguat, a soluble guanylate cyclase (sGC) stimulator, reduces myocardial ischemic reperfusion injury (MIRI), and to learn how this reduction happens.

**Methods and results:**

To develop an ischaemia/reperfusion (I/R) model, the left anterior descending artery was blocked in minipigs under anesthesia for 90 minutes, followed by 180 minutes of reperfusion. Vericiguat is administered three hours before surgery. Two weeks after receiving therapy, pigs underwent cardiovascular magnetic resonance imaging (MRI) to evaluate the results. The MRI results suggest improvement in the myocardial infarct after vericiguat treatment. Vericiguat treatment for two weeks enhanced vascularity, inhibited pro-inflammatory cells, and decreased collagen deposition in the infarct zone of pigs. Short-term experiments investigating possible explanations have indicated that vericiguat has antiapoptotic effects on cardiomyocytes and increases levels of autophagy.

**Conclusions:**

Vericiguat, an SGC activator, reduces MIRI in pigs by boosting autophagy, preventing apoptosis, and promoting angiogenesis.

## Introduction

Restoring myocardial blood supply after acute myocardial infarction by percutaneous coronary intervention, coronary artery bypass grafting, and thrombolytic therapy remains the most effective treatment for saving the dying myocardium, reducing and maintaining cardiac function, and reducing post-infarction myocardial remodelling [1]. However, several studies have shown that MIRI is an unavoidable pathological change in the process of restoring blood perfusion, which can lead to myocardial dysfunction, structural damage, and disturbance of myocardial electrical activity, further aggravating myocardial necrosis. Patients often suffer from serious complications such as arrhythmia, decreased ventricular function, and even sudden death [2, 3].

The heart regularly engages in the autophagic process. Recent studies have discovered that autophagy is an essential process in homeostatic cells to maintain the homeostasis of this interior environment and plays a crucial role in myocardial I/R damage. Numerous autophagic vesicles exist, but the fusion and approval of lysosome-dependent autophagosomes with lysosomes is reduced, ultimately causing the aggravation of myocardial injury. Within the myocardial ischemic phase, activated autophagy can remove misfolded proteins and necrotic mitochondria that induce cardiomyocyte death, etc., and protect the myocardium; within the reperfusion phase, over-activated autophagy forms [4, 5].

Patients with lower ejection fraction (EF) heart failure are seeing better outcomes because to vericigua, a novel orally bioavailable soluble guanylate cyclase stimulator. High-risk heart failure patients on vericiguat had a decreased incidence of mortality from cardiovascular causes or hospitalization for heart failure compared to those taking placebo in the VICTORIA trial [6]. Recently, Yun Cai et al. have shown that vericiiguat can protect against myocardial ischemia-reperfusion injury by improving microcirculation [7]. MIRI may be affected by the au-tophagic pathway, however it is not yet known if this is the case.

Considering that patients require medication before or during PCI and that myocar dial tissue is generally hypoxic, sGC reduction is more common. The purpose of this research was to confirm that the sGC stimulator verici-iguat reduces the damage caused by ischemia and reperfusion to the heart.

## Materials and methods

### Animals

The Laboratory Animal Centre at NanTong University, China, provided six-month-old Chinese miniswines (25±3) kg. Each animal received humane care under the recommendations of the NIH’s Guide for the Care and Use of Laboratory Animals. The Animal Ethics Committee at China’s Nantong University gave its stamp of approval to all of the experiments. Using a random number generator, 28 Chinese miniswine were split into 4 groups: Sham, Sham+vericiguat, I/R, and I/R+vericiguat (The Sham group and the I/R group as control groups were gavaged with PBS, respectively). Anaesthetized minipigs (pro-pofol 0.15 mg/kg/min IV infusion, ketamine 5 mg/kg intramuscular) were subjected to a 90-minute period of left anterior descending artery occlusion followed by 180 minutes of reperfusion to create an ischaemia/reperfusion model. The left anterior descending artery of sham animals was encircled with a suture but not ligated after a thoracotomy was performed. Our previous research suggests that an intragastric dose of 3 mg/kg vericiguat given 3 hours prior to surgery is safe and effective. At 180 min post I/R, animals were euthanized with anesthesia overdose and intravenous potassium. The animal study protocol was approved by the Laboratory Animal Ethics Committee of Nantong University (protocol code P20220224-017).

### CMR protocol and image evaluation

All standard CMR imaging analyses were conducted blind to patient identity and time point assignment by two observers with more than five years of expertise in cardiology diagnostic imaging. Cine imaging at the end of systole and diastole to identify endocardial and epicardial borders, the volumes, mass, and function of the left ventricle were assessed with the software cvi42 (Circle Cardiovascular Imaging Inc., Calgary, Canada). As a diagnostic cutoff for myocardial infarction (MI), the extent of Enhancement with late gadolinium in the myocardium, including the MVO zone, was evaluated. By choosing the slice with the highest signal strength in the infarct region enhancement and labeling sites of interest, we were able to establish cutoffs for increased and non-enhanced cardiac activity, such as the infarct area and normal myocardium. After infarct area recognition was conducted semi-automatically and visually checked for correctness, any discrepancies were fixed by hand.

### Measuring of cyclic guanosine monophosphate (cGMP) concentration

In vitro tissue cGMP levels were determined using a cGMP Direct Immunoassay Kit (Abcam, Cambridge, MA, USA).

### Pathological examination

Histological protocols called for the ventricular tissue to be formalin-fixed and paraffin-embedded. Five millimeter thick tissue sections were taken. To ascertain the extent of fibrosis, Subsequently, hematoxylin and eosin (HE) and Masson’s trichrome stains were applied to the sections. Five millimeters of tissue was formalin-fixed and paraffin-embedded, immunohistochemical examinations were conducted. Tissue was sectioned to a thickness of 5 mm before being formalin-fixed and paraffin-embedded. On the instrument, specimens were deparaffinized and antigen was extracted. All slides were incubated with CD68 (ab955, Abcam, Cambridge, MA, USA), CD31 (ab24590, Cambridge, MA, USA), or CD16 (ab246222, Abcam,Shanghai, China) primary antibodies for 16 minutes, Tissue was sectioned to a depth of 5 mm before being formalin-fixed and paraffin-embedded. The sections were then immersed for eight minutes in primer (anti-rabbit and anti-mouse). The avidin-biotin complex method was used to visualise antibody binding in accordance with the guidelines provided by the maker (Vectastain ABC; Vector). As a negative control, These methods did not include the use of a primary antibody. HE was then used to counterstain the sections.

### Immunofluorescence

Anti-α-SMA primary antibody (ab7817, Cambridge, MA, USA) was utilised to monitor blood vessel formation. After staining the nuclei with DAPI (Vector Laboratories, Burlingame, CA, USA), we followed the manufacturer’s recommendations and treated the sections with fluorescent dye-conjugated secondary antibodies. We used a TUNEL kit (Roche Applied Science; Indianapolis, IN, USA) to investigate cell apoptosis further. A minimum of five randomly selected fields per vein were examined to determine the percentage of TUNEL-positive cardiac cells, and each group had four hearts assessed. Three samples per heart were assessed, and each group had five hearts assessed. The number of α-SMA-positive vessels in each sample was counted by examining three to five randomly selected fields from each specimen.

### Western blotting

In an extraction buffer comprising 50 mM Tris-HCl (pH 7.5), 5 mM EDTA, 10 mM EGTA, 10 mM benzamidine, 0.3% -mercaptoethanol, and 1 X Complete Mini EDTA-Free Protease Inhibitor Cocktail (Roche, Mannheim, Germany), frozen myocardium samples (50–100 mg) were minced and homogenised. The homogenates were subjected to a 10-minute centrifugation at 1000 xg. In order to determine the precise concentrations of proteins in the supernatant, a BCA protein assay kit was utilised. Sodium dodecyl sulphate (SDS) loading buffer was then diluted into the protein samples at a 1:4 ratio. Electrophoretically separated proteins in the range of 20–50 g were transferred to a polyvinylidene difluoride membrane (Millipore, Billerica, MA) using an 8–15% SDS polyacrylamide gel. Before being treated with primary antibodies overnight at 4 degrees Celsius, 5% bovine serum albumin and Tris-buffered saline (TBS) were used to block the membranes for 2 hours. Three 15-minute washes were performed on the membranes. in TBS after being overnight at 4°C incubation with the main antibody. To identify individual proteins, we used the manufacturer’s recommendations for enhanced or super chemiluminescence (Invitrogen, Carlsbad, CA). The Quantity One (Bio-Rad) software measured the relative band intensity.

#### Primary antibodies

Anti-caspase 3 (Cell Signaling, Danvers, MA, United States), anti-Beclin-1, anti-light chain 3 (LC3) and anti-β-ctin(Novus Biologicals, Littleton, CO, United States).

#### Secondary antibodies

Goat anti-mouse IgG (H + L) and goat anti-rabbit IgG (H + L) antibodies, both HRP-conjugated (JacksonImmunoResearch Laboratories, USA).

## Results

### Vericigua reduces myocardial injury

**Fig 1:**
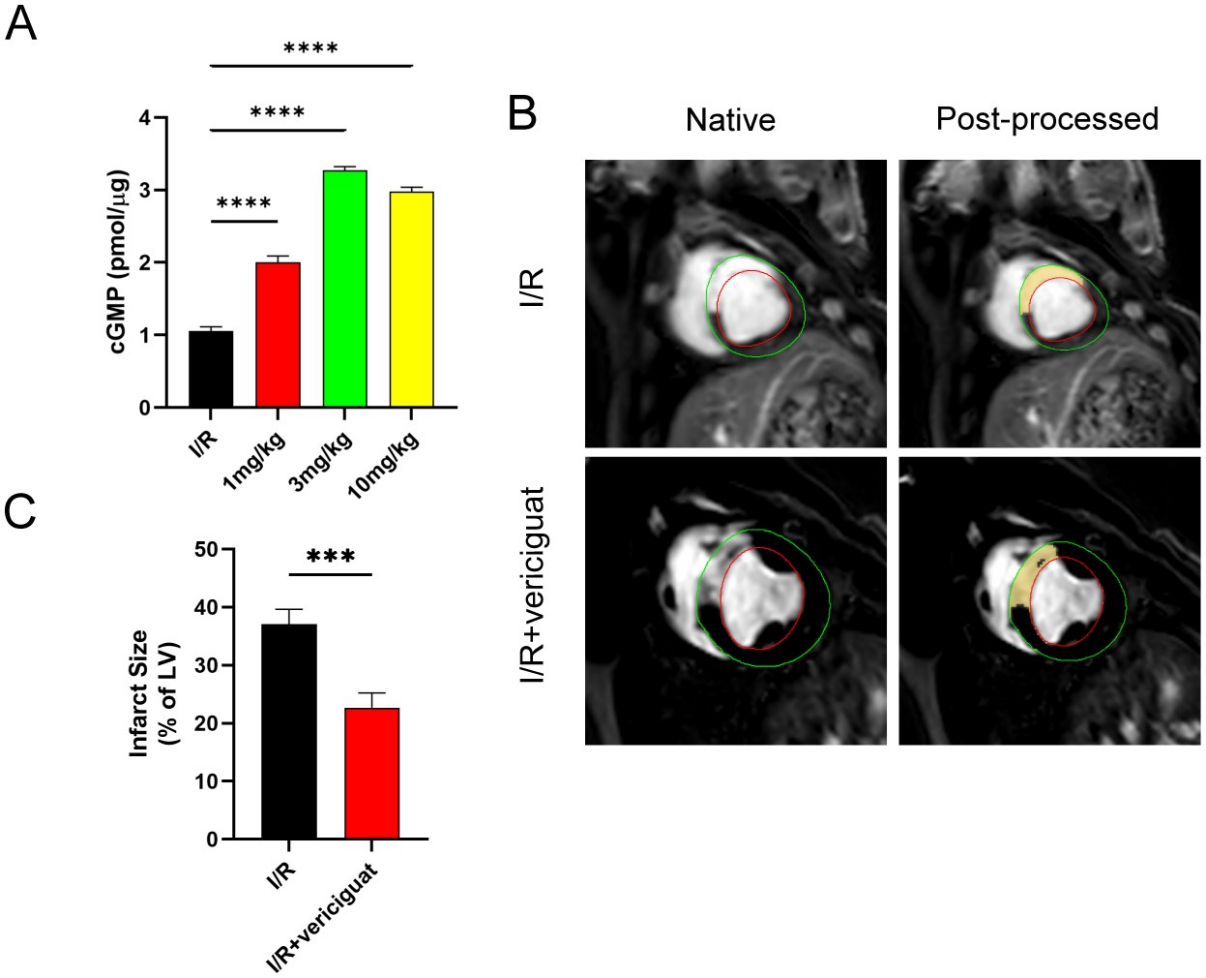
Vericiguat’s impact on infarct size during myocardial ischemia-reperfusion injury. **(A)** Analysis of cGMP Response to Varying Concentrations of Vericiguat. N = 4.**(B, C)** Left ventricular (LV) mass and volume tracing was performed using CMR42 software (Circle Cardiovascular Imaging Inc.), the images were analysed. Infarct areas were designated as DEMRI-enhanced regions. N = 4. Data represent the mean ± SD (nsP > 0:05, *P < 0:05, **P < 0:01, ***P < 0:001,and****P < 0:0001).

The vericiguat medication concentration used in the study was first established. Patients with heart failure and low ejection fraction were evaluated for the safety of vericiguat 10 mg once daily in the VICTORIA trial. It has not been determined in clinical practise whether or not other doses are safe for patients to take. The concentrations used in animal models, which are usually 3 to 10 mg/kg, are essentially safe. In pigs, the effect of vilisicam on cGMP was evident at a concentration of 3 mg/kg (Fig 1A). Therefore, we decided on a 3 mg/kg concentration for the subsequent trial.

For regional scar development and myocardial fibrosis, we examined the existence of late gadolinium enhancement and the proportion of afflicted myocardium. Compared to the I/R group, Vericigua-injected pigs had less regional late gadolinium enhancement in their myocardium 7 days after I/R (Fig 1B and 1C), showing that Vericigua treatment considerably slows the course of myocardial necrosis after I/R.

**Fig 2:**
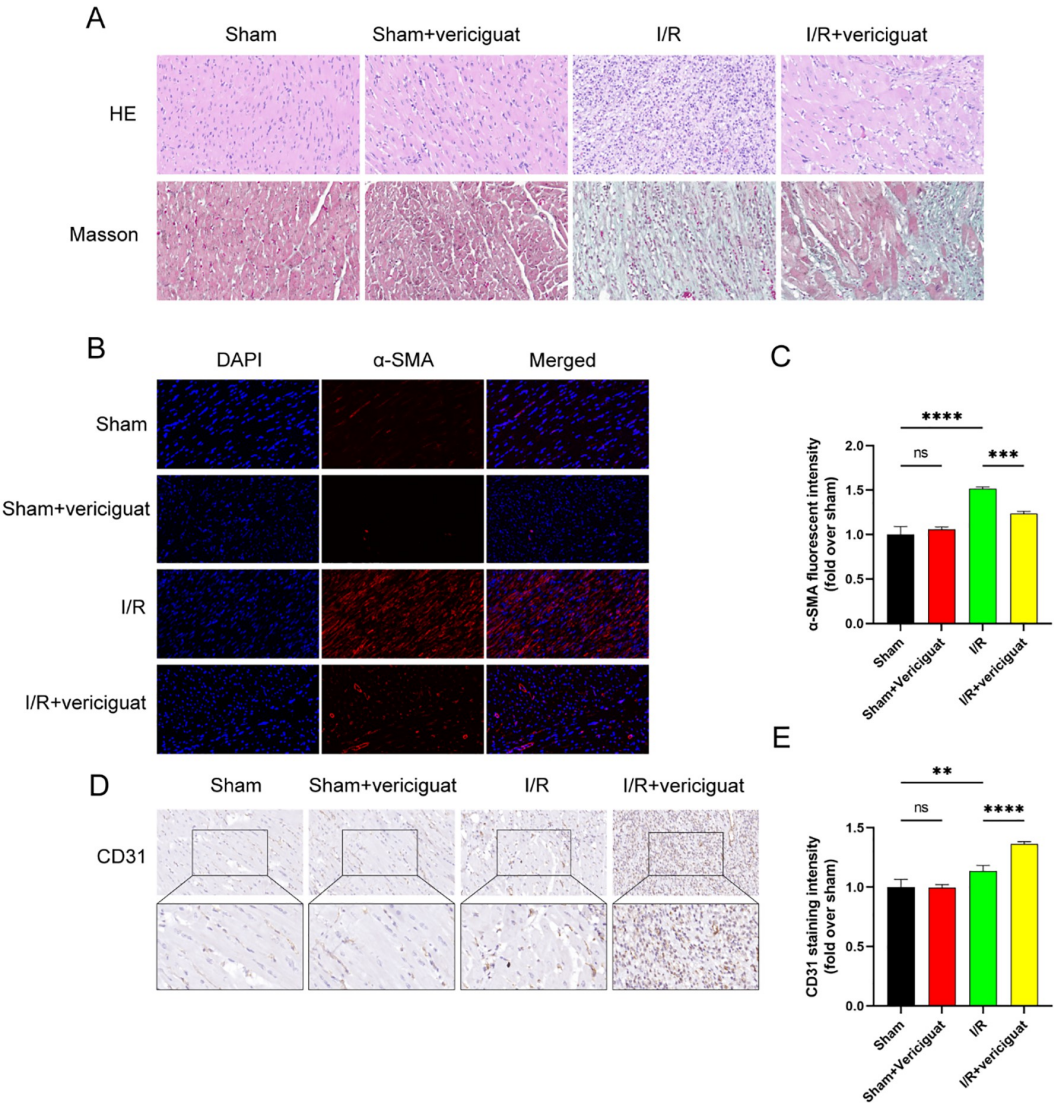
Ischemia-reperfusion injury: Histologic evaluation of sample myocardial sections. **(A)** Vericiguat treatments lessen fibrosis in pig hearts, as seen by Masson’s trichrome staining. Hematoxylin and eosin staining of heart tissue, representative of the whole. The samples were looked at under a light microscope with a magnification setting of x200. N = 4. Pigs were sacrificed 4 weeks after receiving an I/R injury, and cardiac tissue was harvested from the infarct location and embedded in paraffin. N = 4.**(B)** The myocardial tissues were subjected to immunofluorescence analysis of α-SMA, a smooth muscle marker. **(C)** Statistical graph of positive expression rate of α-SMA‐positive cells. **(D)** The myocardial tissues were subjected to immunohistochemistry analysis of CD31, an endothelial cell marker. N = 4.**(E)** Statistical graph of positive expression rate of CD31‐positive cells. Data represent the mean ± SD (nsP > 0:05, *P < 0:05, **P < 0:01, ***P < 0:001,and****P < 0:0001).

Heme-eosin staining of heart sections 7 days after reperfusion also revealed that ventricular morphology was maintained in pigs treated with 3 mg/kg Vericigua, while the number of inflammatory and necrotic cell foci was significantly increased in the PBS group. In contrast, the PBS group saw a marked increase in the frequency of inflammatory and necrotic cell foci (Fig 2A). Vericigua was also found to decrease collagen deposition, as seen by Masson staining, in these same tissue sections (Fig 2A).

The subsequent evaluation of the expression of α-SMA was carried out with the purpose of providing more insight into the potential mechanism through which Vericigua inhibits fibrosis. A series of immunofluorescence experiments were carried out. The expression of α-SMA was shown to be significantly higher in the group that had myocardial infarction compared to the group that had sham surgery, and those who were exposed to Vericigua showed notably diminished levels. (Fig 2B and 2C).

Meanwhile, the results of immunohistochemical analysis showed that CD31-positive areas were elevated in the hearts of mice receiving Vericigua (Fig 2D and 2E). These findings suggest that Vericigua improves the function of porcine infarcted hearts, reduces infarct size, and promotes angiogenesis.

### Vericiguat inhibits the activation of pro-inflammatory macrophages

In response to cardiac injury, the inflammatory cascade is critical, yet excessive inflammatory reactions hinder heart repair. Inflammatory gene overexpression is a hallmark of M1 macrophages, a proinflammatory subtype.

**Fig 3:**
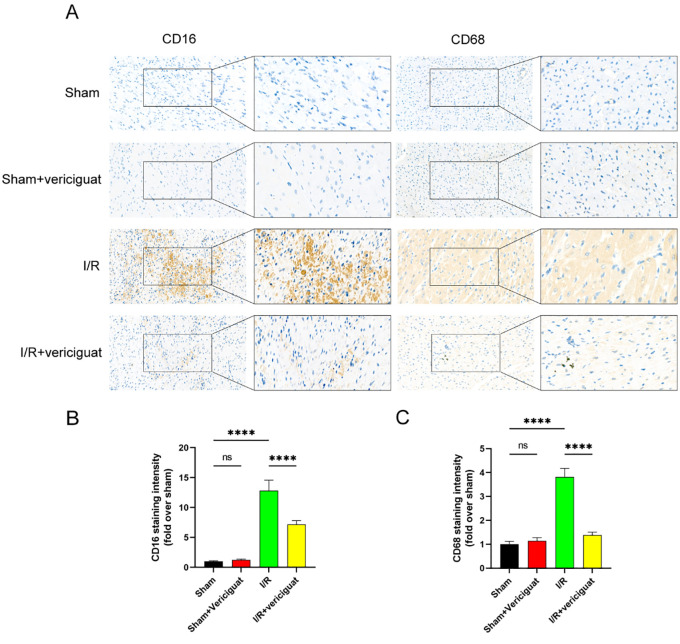
Inhibition of vericiguat on CD16 and CD68 expression. **(A)** Representative photomicrographs of CD16 and CD68 staining (scale bar: 50 μm). N = 4.**(B)** Quantitative analysis of CD16 (+) area.**(C)** Quantitative analysis of CD68 (+) area. Data represent the mean ± SD (nsP > 0:05, *P < 0:05, **P < 0:01, ***P < 0:001,and****P < 0:0001).

It was unclear whether or not vericiguat had an anti-inflammatory effect. To identify the phenotype of cardiac macrophages, we stained macrophage biomarkers (CD68) and M1 biomarkers (CD16) with specific antibodies. Our results demonstrated that the vericiguat-treated group had fewer CD68+ and CD16+ macrophages than the I/R group (Fig 3A–3C). This suggests that vericiguat inhibits the activation of M1-type macrophages.

### Treatment with Vericigua inhibits I/R-induced apoptosis

**Fig 4:**
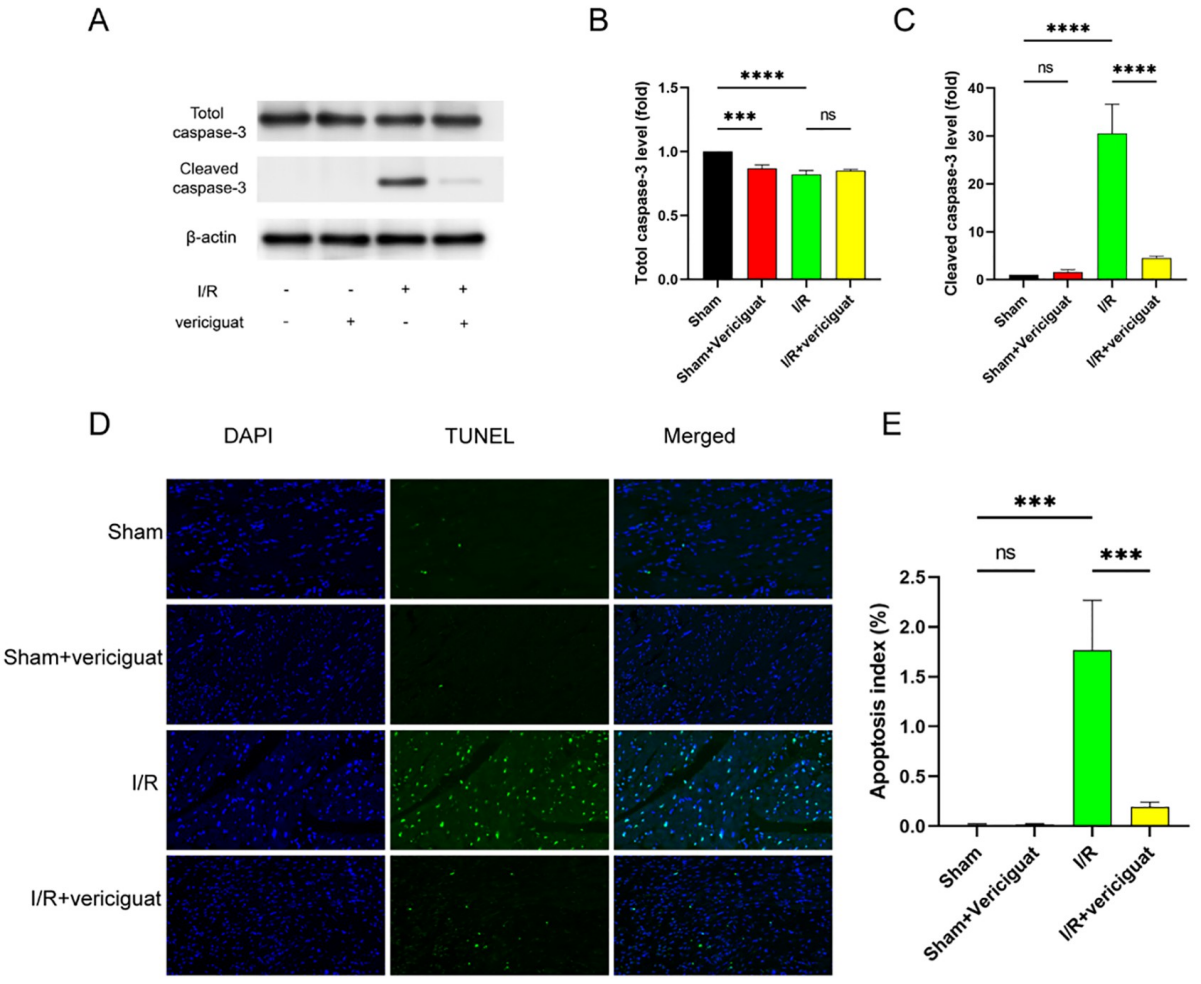
Apoptotic activity following I/R damage and vericigua therapy. **(A)** Western blot examination of cardiac tissue at 24 hours post-I/R injury reveals caspase-3 cleavage. **(B, C)** Cleaved caspase-3 and Totle-caspase-3 were analysed by Western blotting. N = 3.**(D)** Vericigua treatments decrease TUNEL positive cells, as evidenced by representative photos of TUNEL staining. **(E)** Summarised TUNEL staining data demonstrates vericigua treatments dramatically decrease infarct-area apoptotic cardiomyocytes 24 hours after I/R injury. N = 3 Data represent the mean ± SD (nsP > 0:05, *P < 0:05, **P < 0:01, ***P < 0:001,and****P < 0:0001).

Many different types of cardiac pathology are associated with apoptosis. After 24 hours of I/R, hearts from control and Vericigua-treated pigs were stained with terminal deoxynucleotidyl transferase-mediated DUTP nick end labelling (TUNEL) to examine the anti-apoptotic effect of Vericigua treatment. TUNEL-positive cells were hardly detectable in the hearts of control or vericigua-treated pigs after sham surgery (Fig 4D), but quantitative analysis showed that the proportion of TUNEL-positive cells in the ischemic zone was significantly lower in Vericigua-treated pigs after I/R injury compared with control pigs (Fig 4E). Myocardial I/R damage induced a rise in cleaved caspase-3, an active form of the pro-apoptotic proenzyme, whereas Vericigua therapy suppressed this rise (Fig 4A–4C).

### Autophagy is involved in the cardioprotective effect of vericiguat

**Fig 5:**
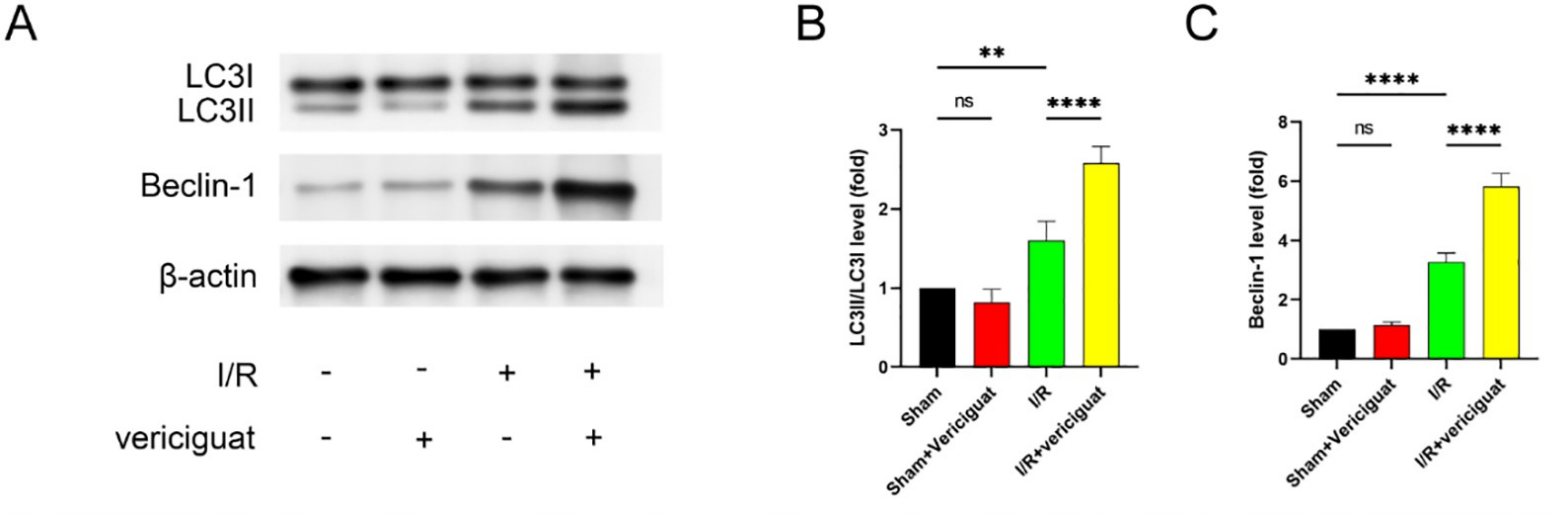
Expression of Beclin-1, LC3-I and LC3-II with vericiguat-pretreatment. **(A)** Original immunoblots. **(B, C)** Immunoreactivity of Beclin-1 and LC3-II/LC3-I, corrected for β-actin expression and normalized to control value. N = 3 Data represent the mean ± SD (nsP > 0:05, *P < 0:05, **P < 0:01, ***P < 0:001,and****P < 0:0001).

Next, we looked into whether autophagy plays a role in Vericigua’s cardioprotective properties. Our findings thus provide qualitative evidence that Vericigua induced an upregulation of Beclin-1 and LC3B-II expression in the heart muscle (Fig 5A–5C).

Beclin-1 and Lc3-II are believed to be unique indicators of autophagy and autophagy formation, and their elevated expression in the presence of Vericigua treatment indicates induction of autophagy.

## Discussion

Few current studies have evaluated the efficacy and feasibility of Vericigua for AMI in preclinical animal models, and this study reproduces the current procedure for the administration of AMI in humans. The pig was chosen by us type of I/R damage due to the similarity between porcine heart anatomy and compared to the center that is individual our capability to utilize The instrumentation that is same is employed in humans [8, 9].

MIRI is a common pathophysiological phenomenon involving myocardial metabolic disturbances and structural remodelling [10]. Previous studies have shown that MIRI is caused by inflammatory responses, microembolism, and cell death, leading to severe cardiovascular dysfunction [11]. Although many treatments, including angioplasty, calcium channel blockers, and anticoagulation, have been tried for MIRI, their effectiveness is hampered by a number of problems [12, 13]. Patients with acute coronary syndrome have lower levels of sGC. Successful reperfusion therapy has been shown to enhance myocardial tissue and function in AMI patients, and this improvement correlates positively with sGC activity. According to the findings, a drop in sGC levels following the onset of AMI may be an indicator of impending cardiac complications. Vericiguat is a novel oral soluble guanylate cyclase stimulator that directly stimulates soluble guanylate cyclase through nitric oxide-independent binding sites and enhances the cyclic guanosine monophosphate (GMP) pathway by stabilising nitric oxide-binding sites and sensitising soluble guanylate cyclase to endogenous nitric oxide [14]. Phosphodiesterases, protein kinases that are cGMP dependent and ion channels that are controlled by cGMP are only a few of the intracellular proteins that cGMP interacts with. These transduction cascades ultimately mediate a wide range of physiological effects, some of which include tissue-protective properties, such as the inhibition and relaxation of smooth muscle growth, the recruitment of leukocytes, and the activity of platelets [15]. Inadequate bioavailability of NO, and thus weaker activation of sGC, and thus lower synthesis of cGMP, has been associated to the aetiology of different disorders, particularly those of the heart [16–20]. Vericigua has been demonstrated to prevent cardiac damage during the I/R response in mice in experimental experiments [7]. Here we show that in pig models, supplementing Vericigua can reduce the area of heart attack and alleviate the response of pig heart muscle to I/R injury. Our findings therefore indicate that Vericigua may offer some protection against MIRI damage.

In eukaryotic species, autophagy is a crucial process for recycling intracellular components. More and more research confirms that autophagy is activated in numerous cardiovascular pathogenic conditions, such as myocardial hypertrophy [21], heart failure [22], atherosclerosis [23], and myocardial I/R injury [24]. Emerging investigations in recent years have confirmed the significance of autophagy in MIRI. However, it is still debatable whether reperfusion-induced changes in autophagy are helpful or harmful, and there is experimental data to support both perspectives [25–28]. Kai-yu Huang et al. found that metformin reduced myocardial I/R injury by inhibiting autophagy levels in a mouse I/R model [29]. Further evidence linking autophagy suppression by Chuanzin Dome to improved cell survival in the I/R model was provided by Zhi Zuo et al. [30] Autophagy enhancer rapamycin significantly enhances autophagy, improves cell viability, and reduces apoptosis in NRVC cells [31]. To a similar extent, Han Xu et al. are discovering that pharmacological stimulation of autophagy corresponds with enhanced survivability, as measured by decreased cellular death in cultured NRVCs following H/R, which may be easily and substantially suppressed by the autophagy inhibitor chloroquine [32]. Vericigua treatment, which may cause autophagy, has been connected with limited infarct size, paid down apoptosis, and reserved function that is cardiac in the setting of I/R damage.

This work may be limited by its focus on a single protective mechanism, namely the impact of acute low-dose Vericigua pretreatment on myocardial I/R damage. But in fact, it seems essential to determine whether Vericigua supplemented during reperfusion has the same or other protective mechanisms related to autophagy. And whether chronic Vericigua treatment affects the alteration of autophagy and thus protects the myocardium may be a topic worth investigating. In addition, by using transgenic mice in place of pharmaceutical inhibitors, we intend to provide more clarification of our theories.

In summary, our study demonstrates for the first time that an acute, single, low-dose oral administration of Vericigua before myocardial ischemia enhances myocardial autophagy. Vericigua, even at low dosages, protects the myocardium against I/R damage by increasing autophagy in the heart, which in turn reduces inflammation and apoptosis.

## Conclusions

SGC stimulator vericiguat ameliorates myocardial ischemia-reperfusion injury in pigs by promoting angiogenesis, enhancing autophagy and anti-apoptosis.

## Supporting information

S1 Raw images (PDF)

S1 Data (ZIP)
