# Supplementary material for: Vericiguat protects against cardiac damage in a pig model of ischemia/reperfusion
Source: PLoS One. 2023 Dec 22;18(12):e0295566. doi: 10.1371/journal.pone.0295566 (PMC10745182; doi:10.1371/journal.pone.0295566)

Sham, Sham+vericiguat, I/R, I/R+vericiguat

43KDA

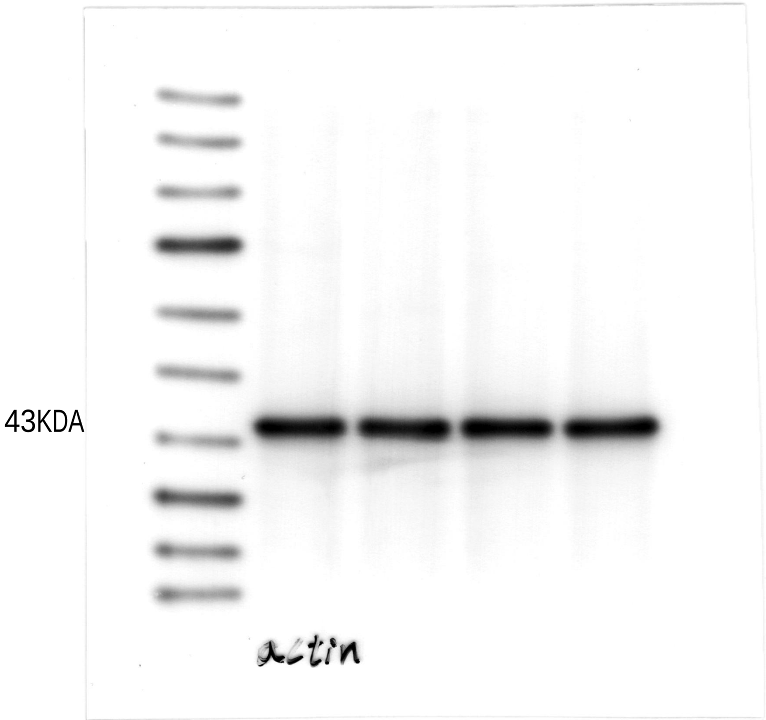

Sham,Sham+vericiguat,I/R,I/R+vericiguat

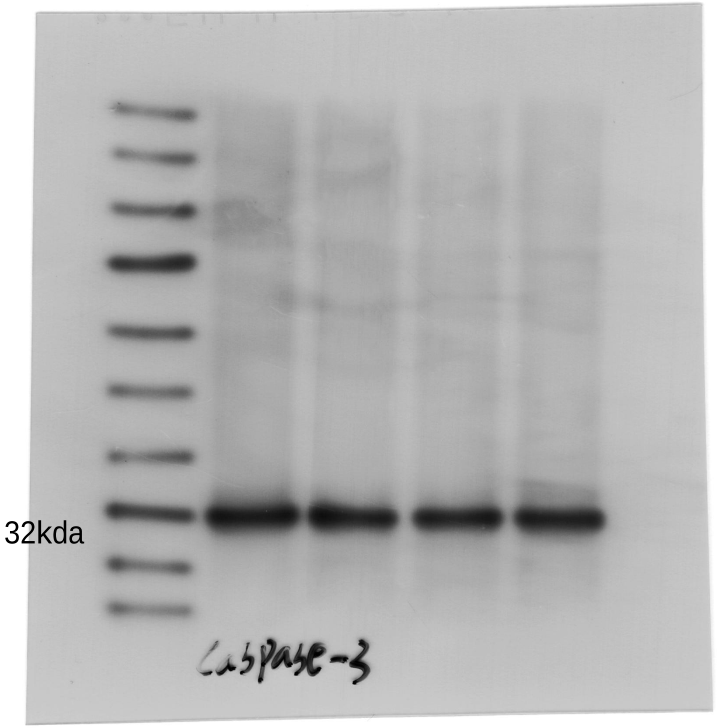

Sham,Sham+vericiguat,I/R,I/R+vericiguat

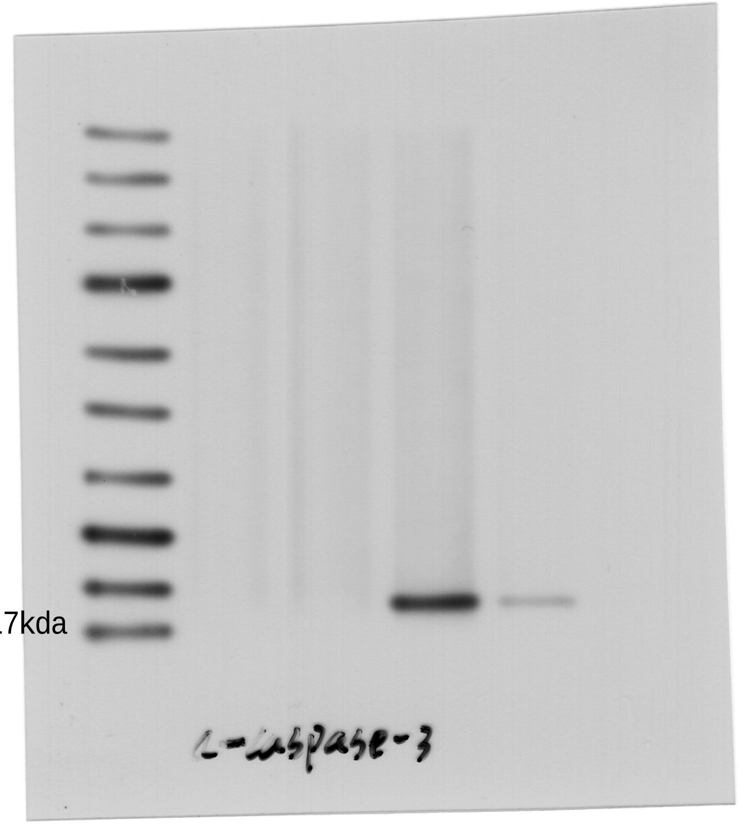


17kda

Sham,Sham+vericiguat,I/R,I/R+vericiguat

43kda

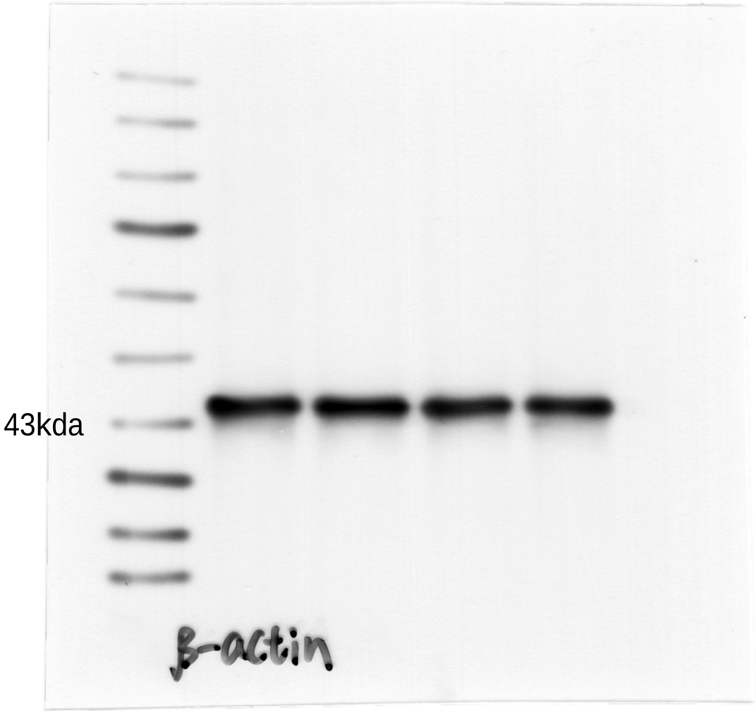

Sham,Sham+vericiguat,I/R,I/R+vericiguat

52KDA

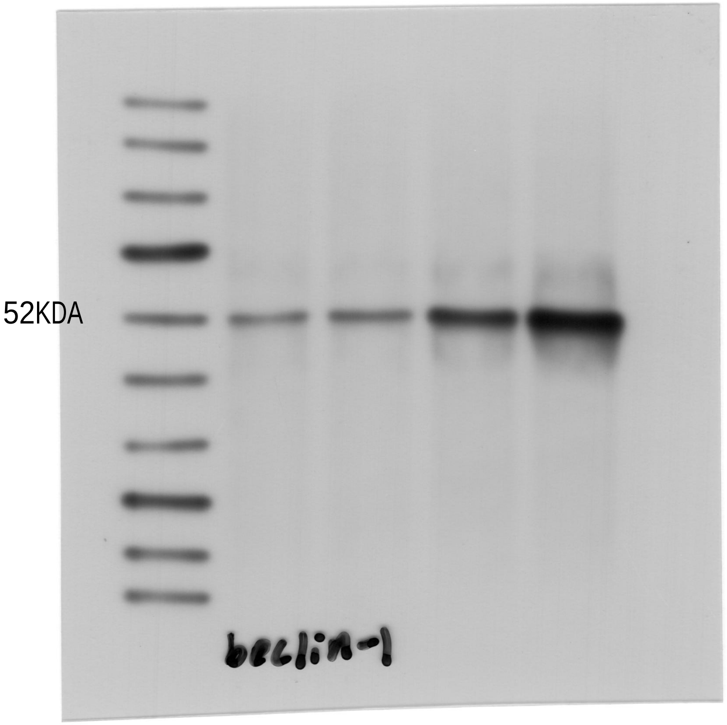

Sham,Sham+vericiguat,I/R,I/R+vericiguat

16KDA
14KDA

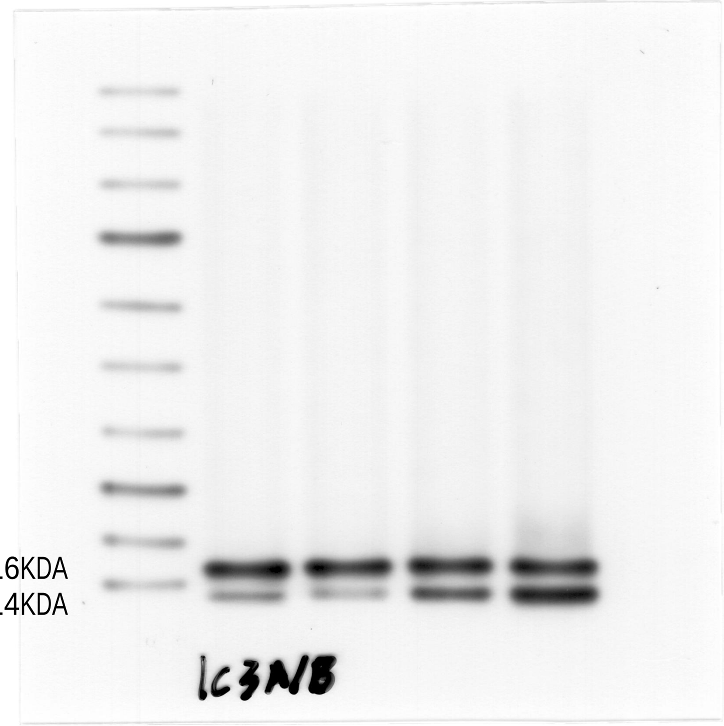

Supplement: S1 Raw images — (PDF) [file pone.0295566.s001.pdf]
