# Supplementary figures and images for: Vericiguat protects against cardiac damage in a pig model of ischemia/reperfusion
Source: PLoS One. 2023 Dec 22;18(12):e0295566. doi: 10.1371/journal.pone.0295566 (PMC10745182; doi:10.1371/journal.pone.0295566)

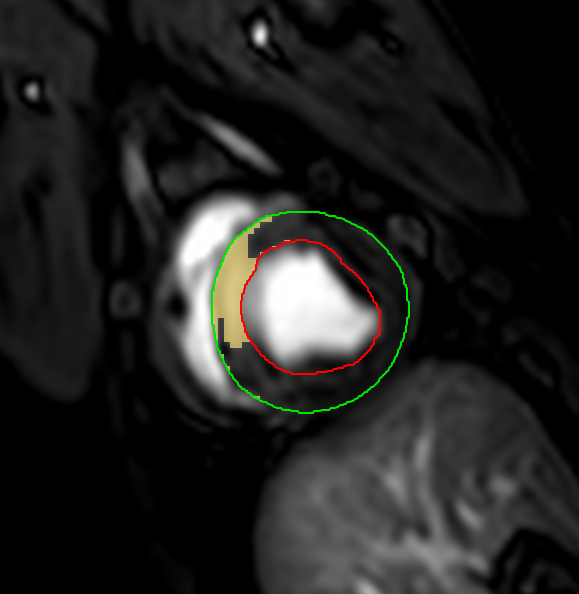

Supplement: S1 Data — (ZIP) [file pone.0295566.s002.zip › minimal underlying data/Fig1/MRI/IR+vericiguat1.tiff]

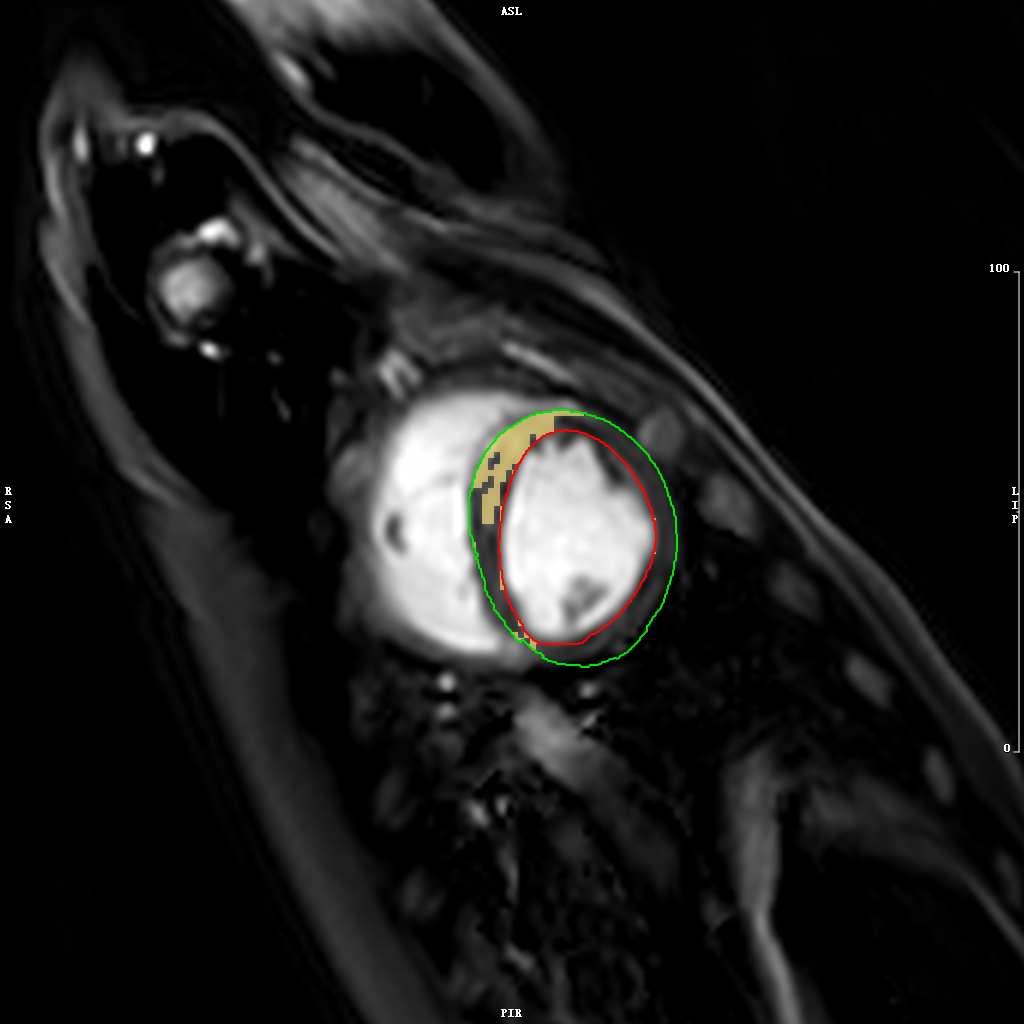

Supplement: S1 Data — (ZIP) [file pone.0295566.s002.zip › minimal underlying data/Fig1/MRI/IR+vericiguat2.tiff]

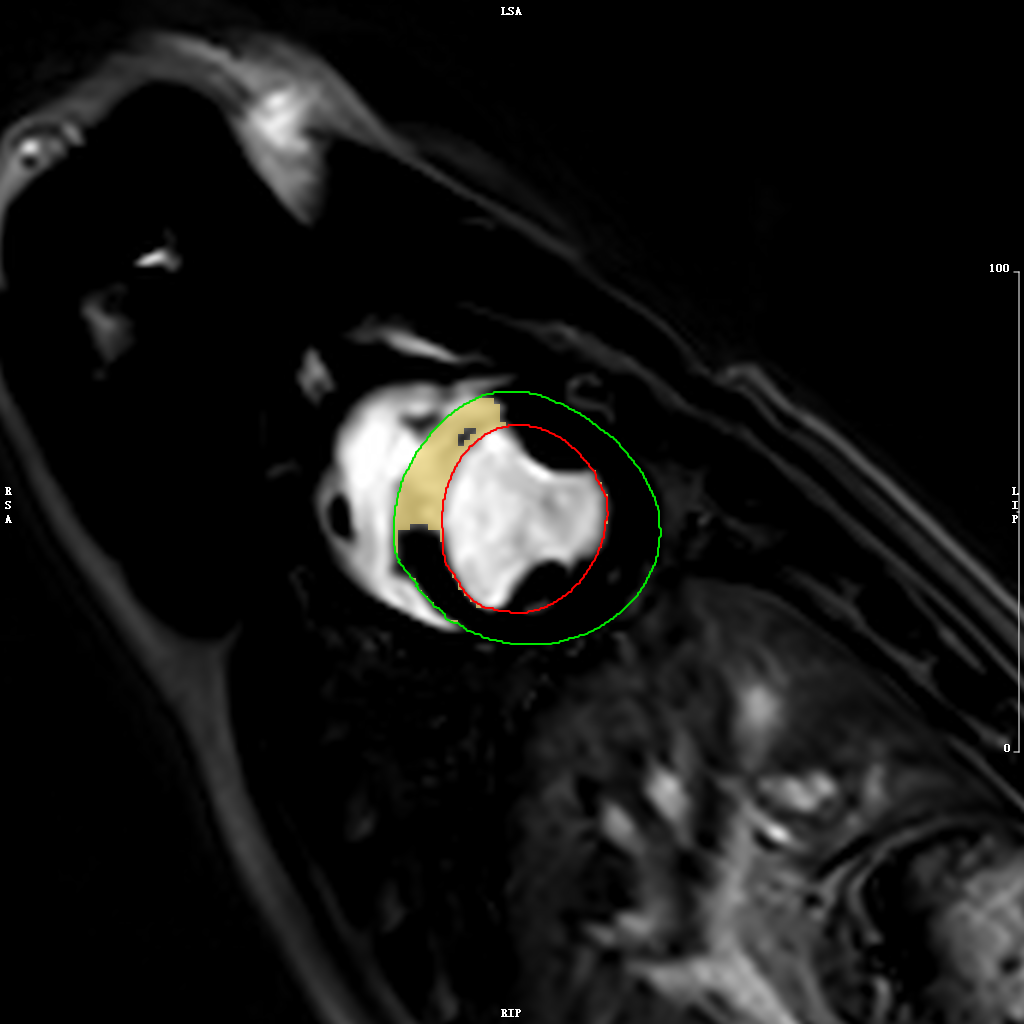

Supplement: S1 Data — (ZIP) [file pone.0295566.s002.zip › minimal underlying data/Fig1/MRI/IR+vericiguat3.tiff]

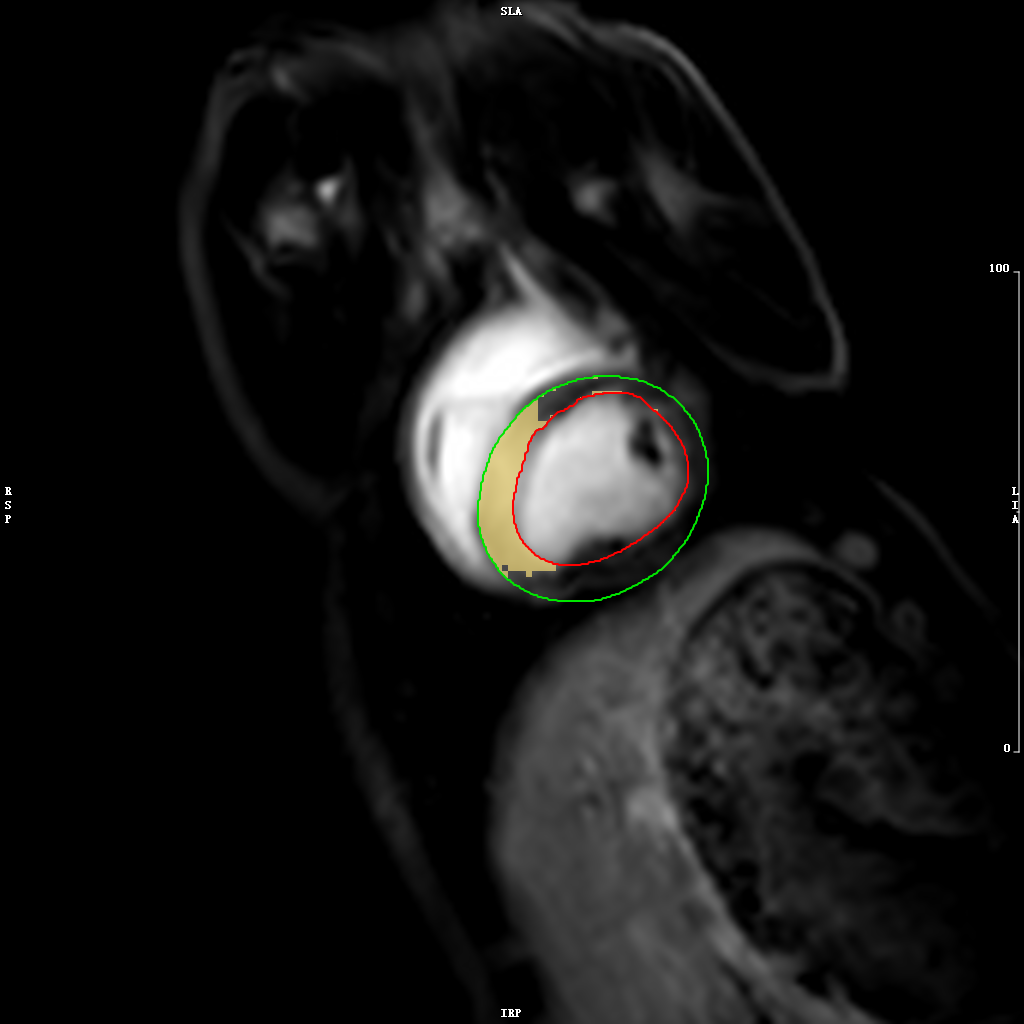

Supplement: S1 Data — (ZIP) [file pone.0295566.s002.zip › minimal underlying data/Fig1/MRI/IR1.tiff]

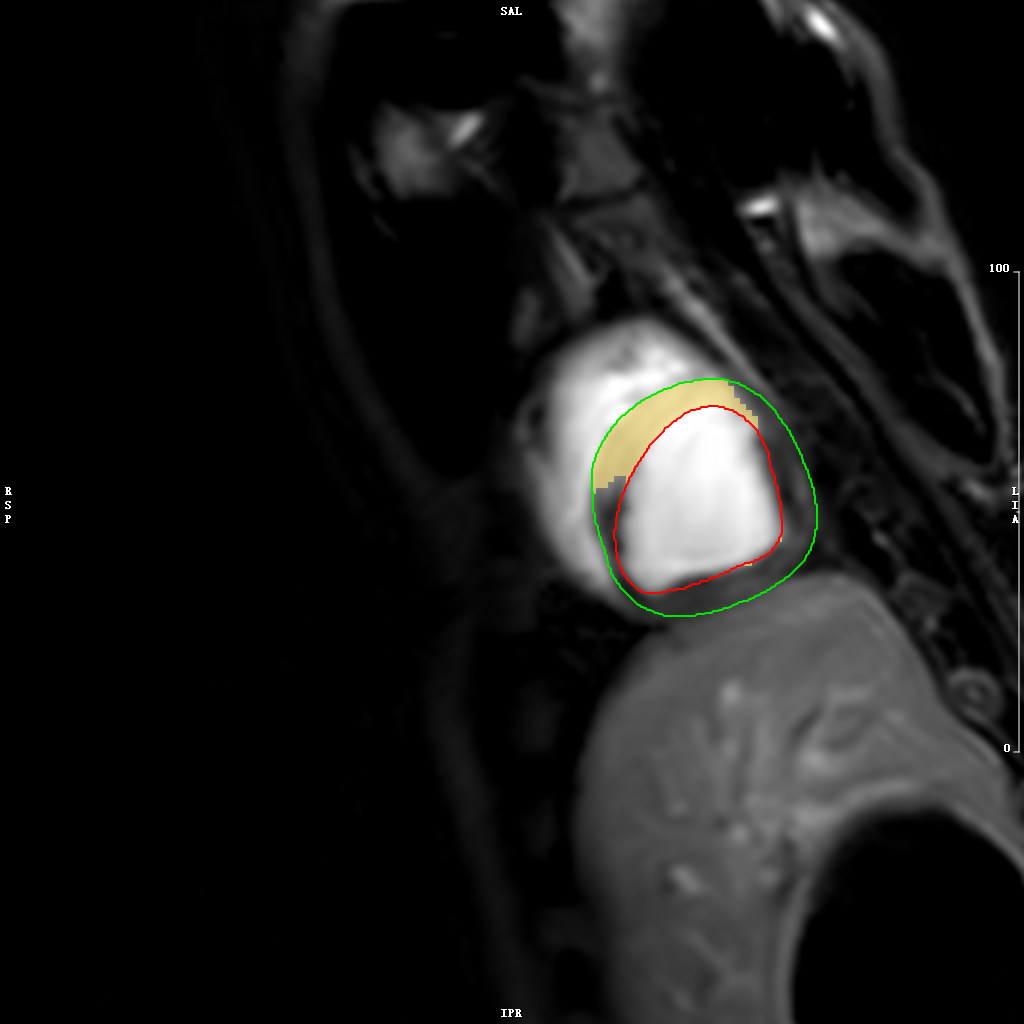

Supplement: S1 Data — (ZIP) [file pone.0295566.s002.zip › minimal underlying data/Fig1/MRI/IR2.tiff]

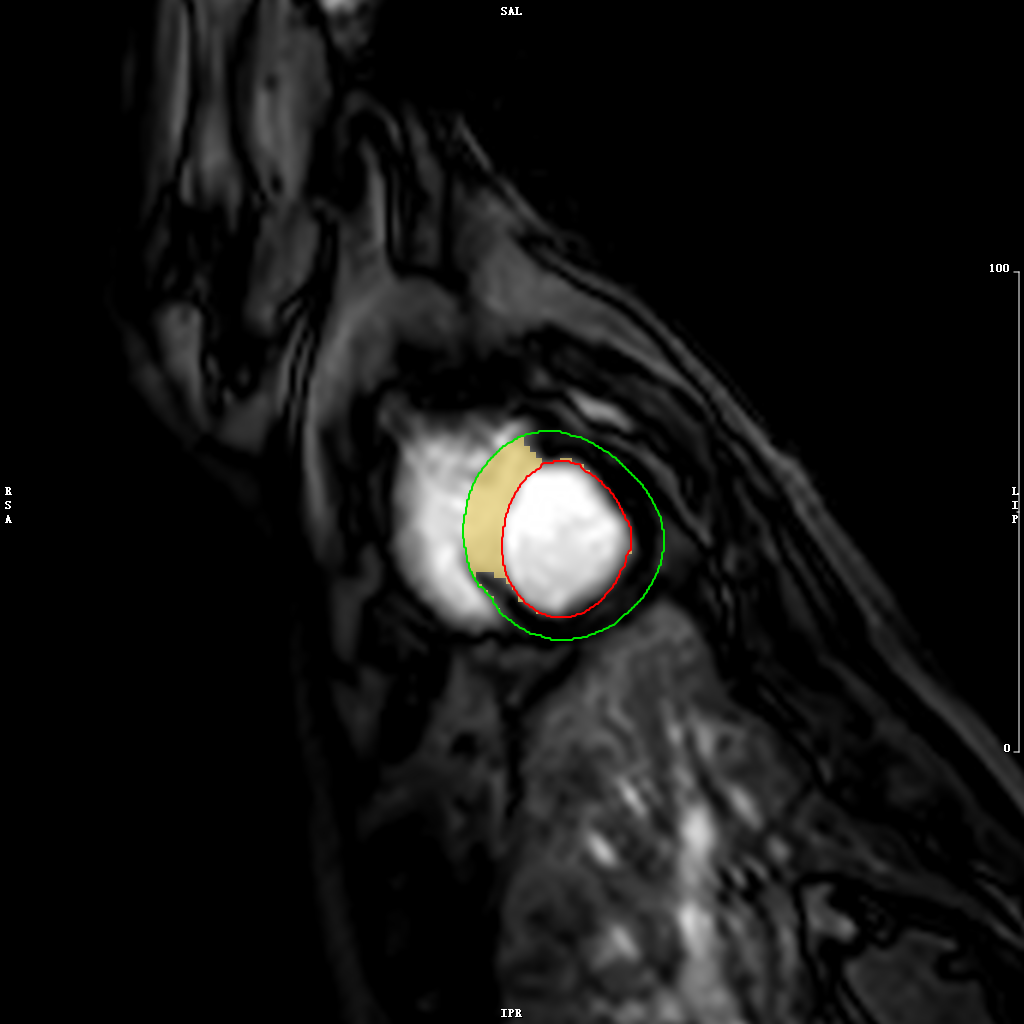

Supplement: S1 Data — (ZIP) [file pone.0295566.s002.zip › minimal underlying data/Fig1/MRI/IR3.tiff]

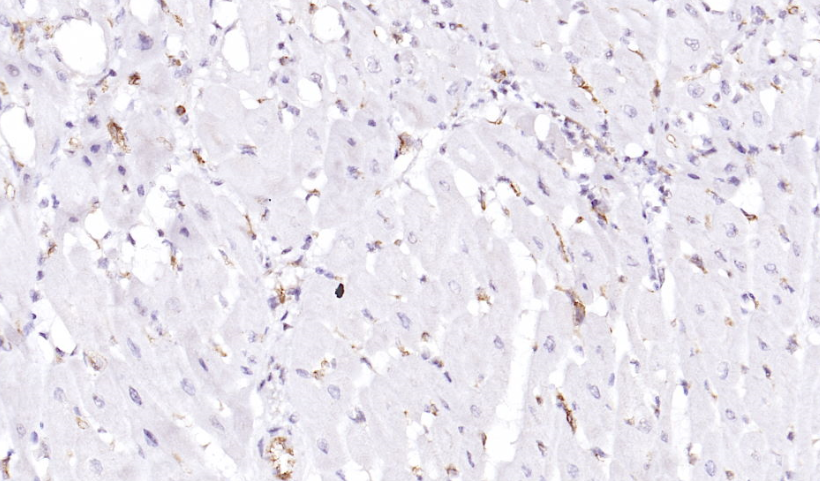

Supplement: S1 Data — (ZIP) [file pone.0295566.s002.zip › minimal underlying data/Fig2/CD31/IR+vericiguat.tif]

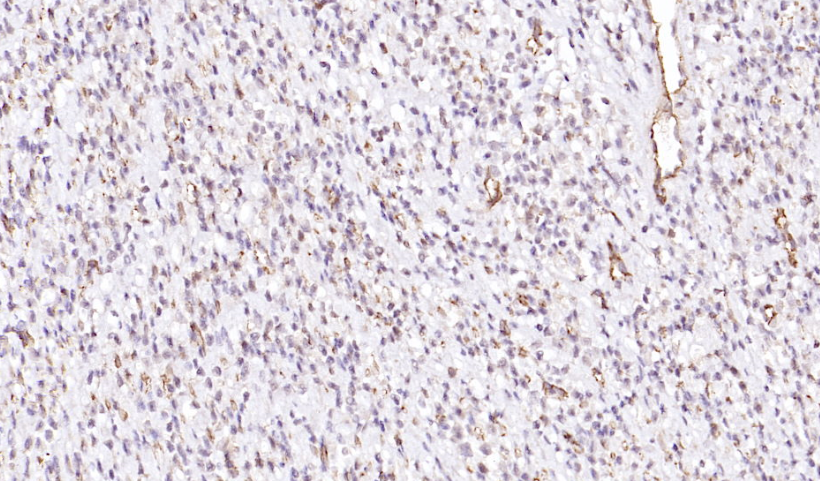

Supplement: S1 Data — (ZIP) [file pone.0295566.s002.zip › minimal underlying data/Fig2/CD31/IR.tif]

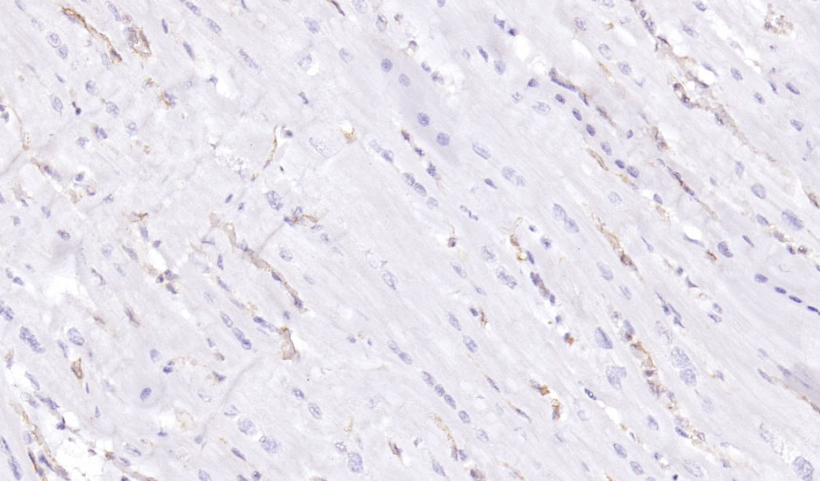

Supplement: S1 Data — (ZIP) [file pone.0295566.s002.zip › minimal underlying data/Fig2/CD31/sham+vericiguat.tif]

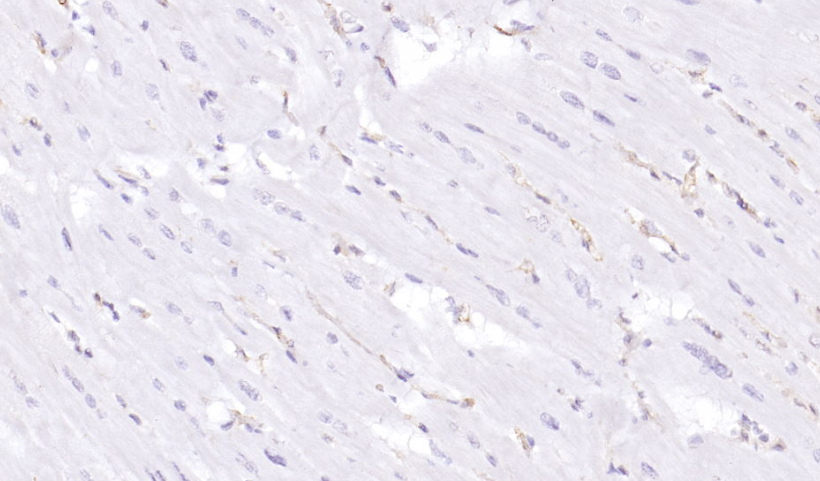

Supplement: S1 Data — (ZIP) [file pone.0295566.s002.zip › minimal underlying data/Fig2/CD31/sham.tif]

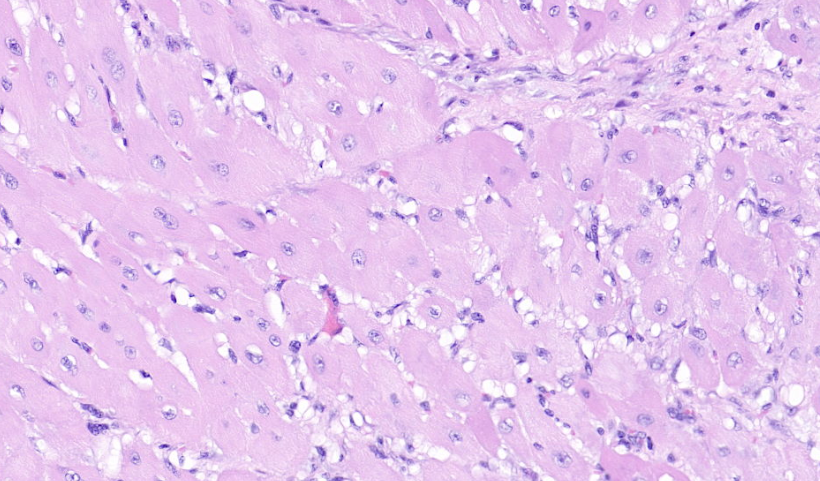

Supplement: S1 Data — (ZIP) [file pone.0295566.s002.zip › minimal underlying data/Fig2/HE/IR+vericiguat.tif]

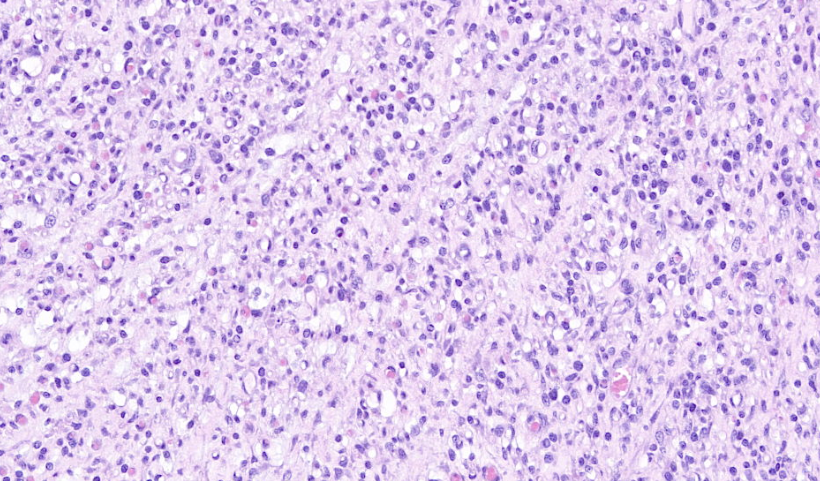

Supplement: S1 Data — (ZIP) [file pone.0295566.s002.zip › minimal underlying data/Fig2/HE/IR.tif]

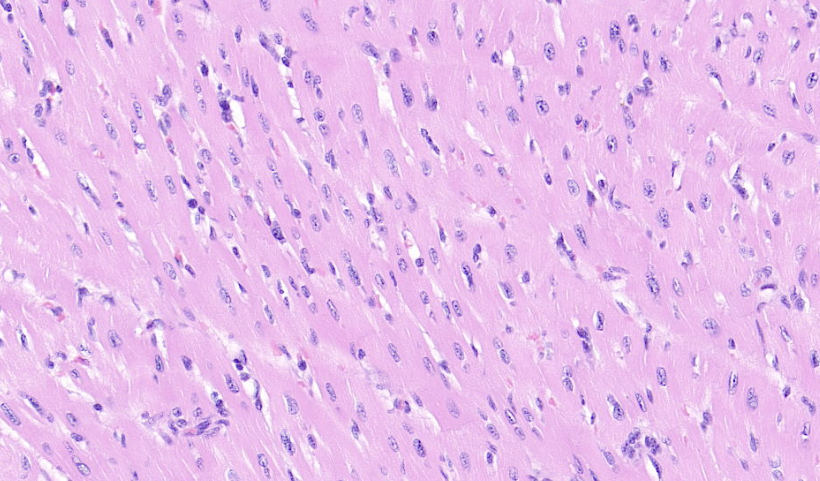

Supplement: S1 Data — (ZIP) [file pone.0295566.s002.zip › minimal underlying data/Fig2/HE/sham+vericiguat.tif]

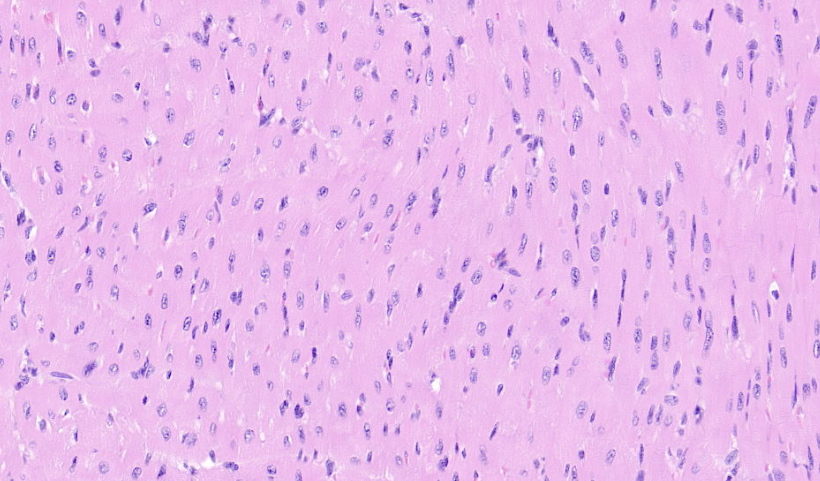

Supplement: S1 Data — (ZIP) [file pone.0295566.s002.zip › minimal underlying data/Fig2/HE/sham.tif]

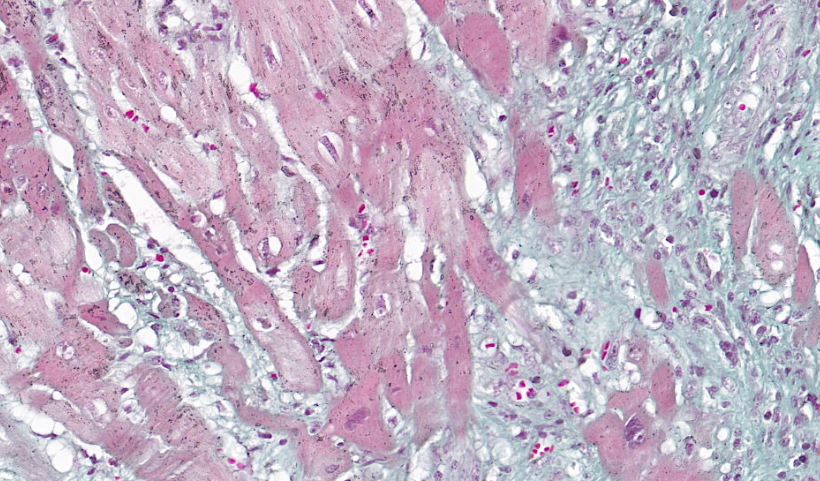

Supplement: S1 Data — (ZIP) [file pone.0295566.s002.zip › minimal underlying data/Fig2/masson/IR+vericiguat.tif]

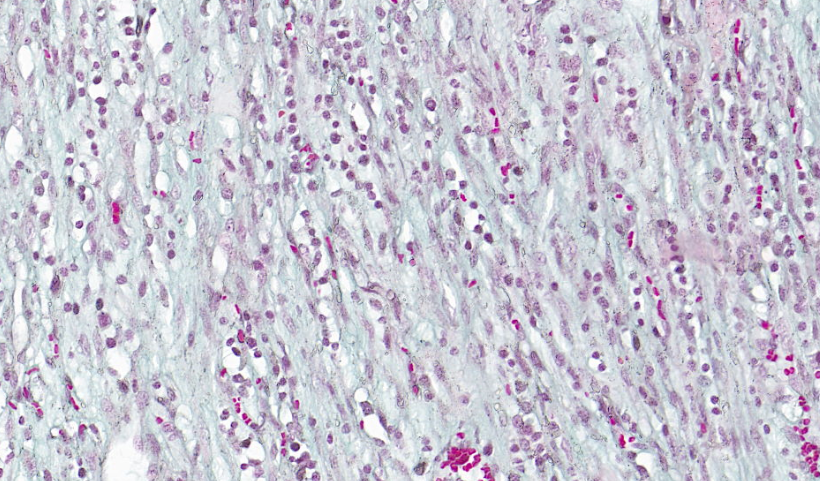

Supplement: S1 Data — (ZIP) [file pone.0295566.s002.zip › minimal underlying data/Fig2/masson/IR.tif]

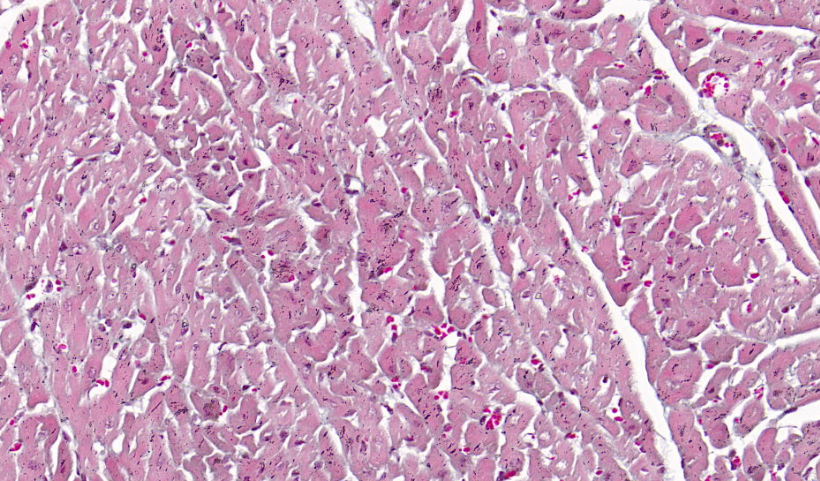

Supplement: S1 Data — (ZIP) [file pone.0295566.s002.zip › minimal underlying data/Fig2/masson/sham+vericiguat.tif]

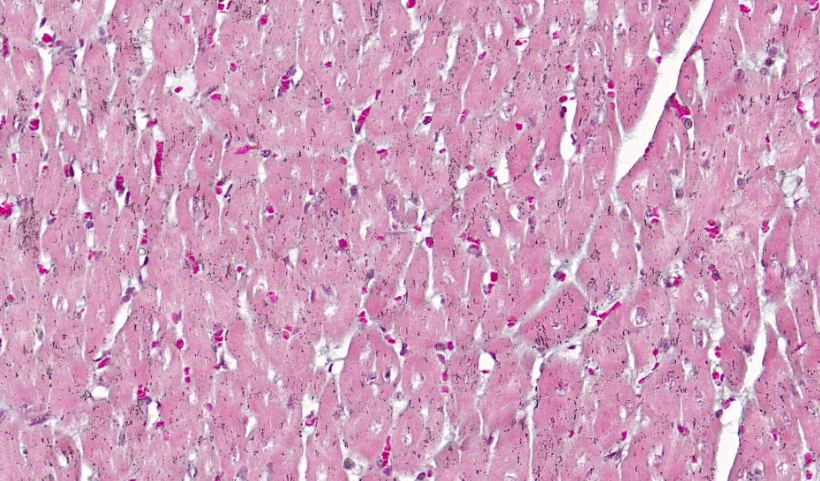

Supplement: S1 Data — (ZIP) [file pone.0295566.s002.zip › minimal underlying data/Fig2/masson/sham.tif]

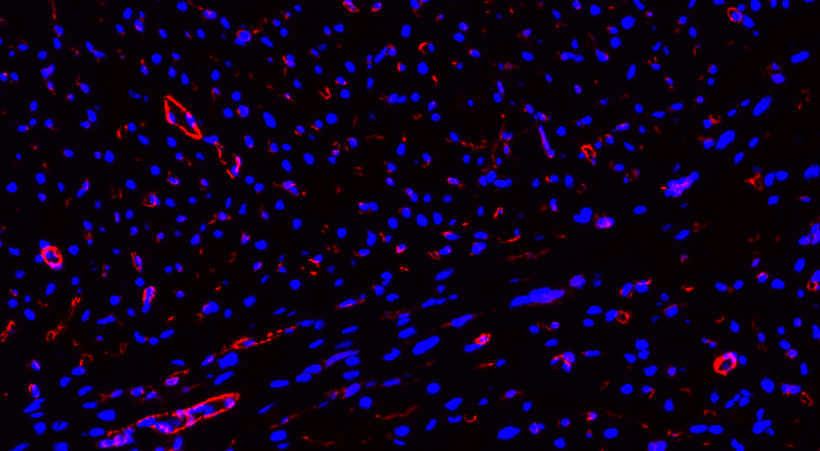

Supplement: S1 Data — (ZIP) [file pone.0295566.s002.zip › minimal underlying data/Fig2/αSMA/IR+vericiguat1.tif]

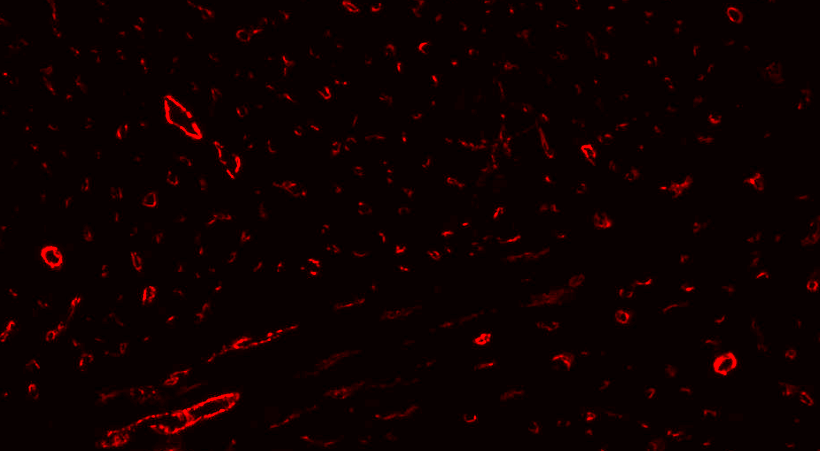

Supplement: S1 Data — (ZIP) [file pone.0295566.s002.zip › minimal underlying data/Fig2/αSMA/IR+vericiguat2.tif]

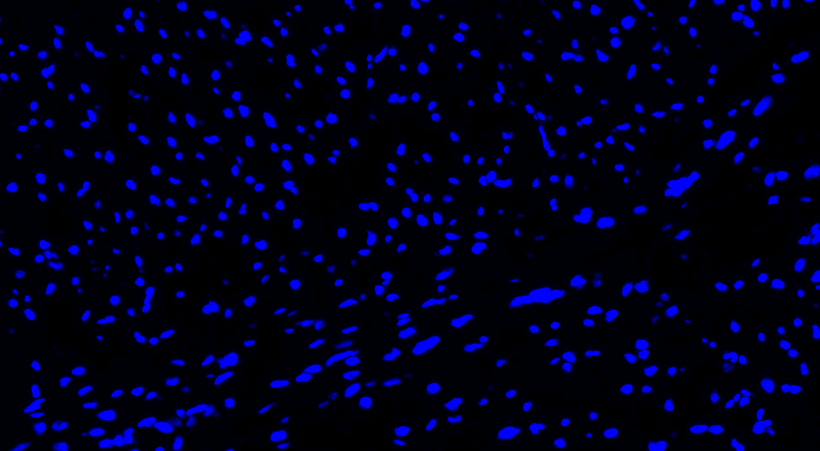

Supplement: S1 Data — (ZIP) [file pone.0295566.s002.zip › minimal underlying data/Fig2/αSMA/IR+vericiguat3.tif]

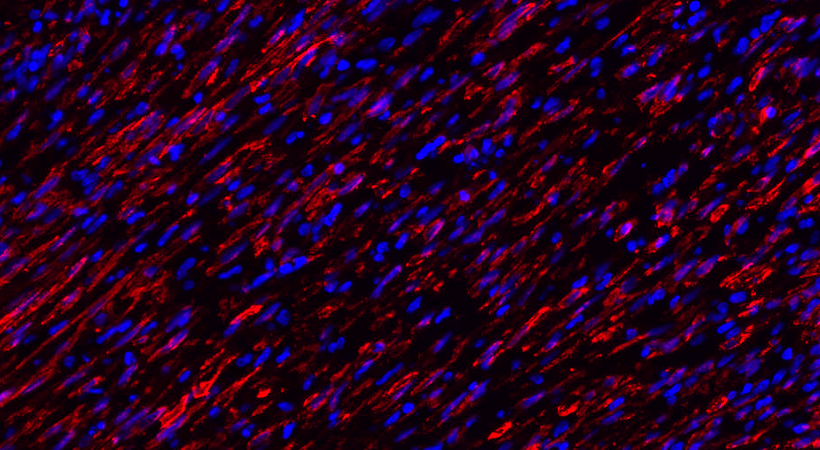

Supplement: S1 Data — (ZIP) [file pone.0295566.s002.zip › minimal underlying data/Fig2/αSMA/IR1.tif]

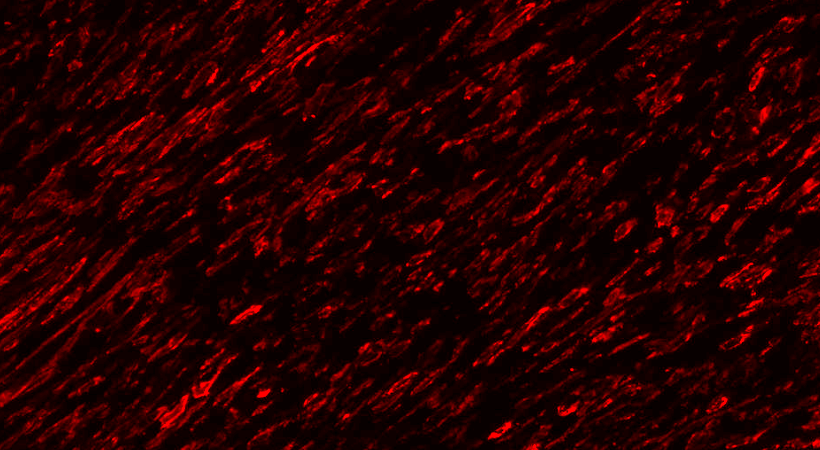

Supplement: S1 Data — (ZIP) [file pone.0295566.s002.zip › minimal underlying data/Fig2/αSMA/IR2.tif]

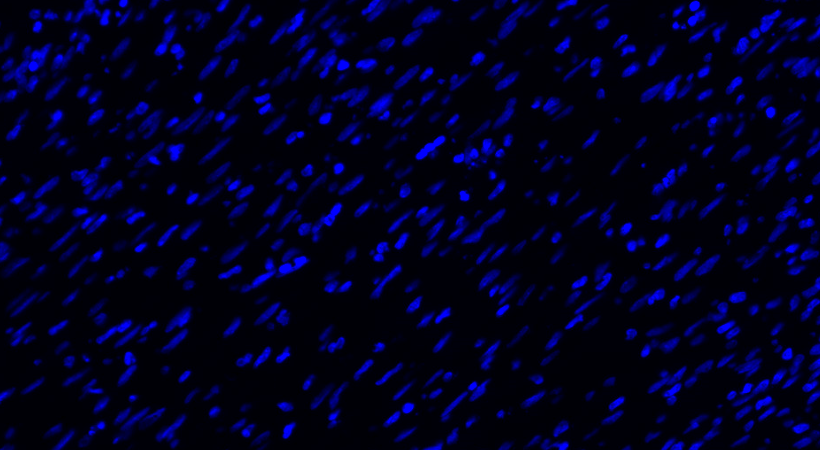

Supplement: S1 Data — (ZIP) [file pone.0295566.s002.zip › minimal underlying data/Fig2/αSMA/IR3.tif]

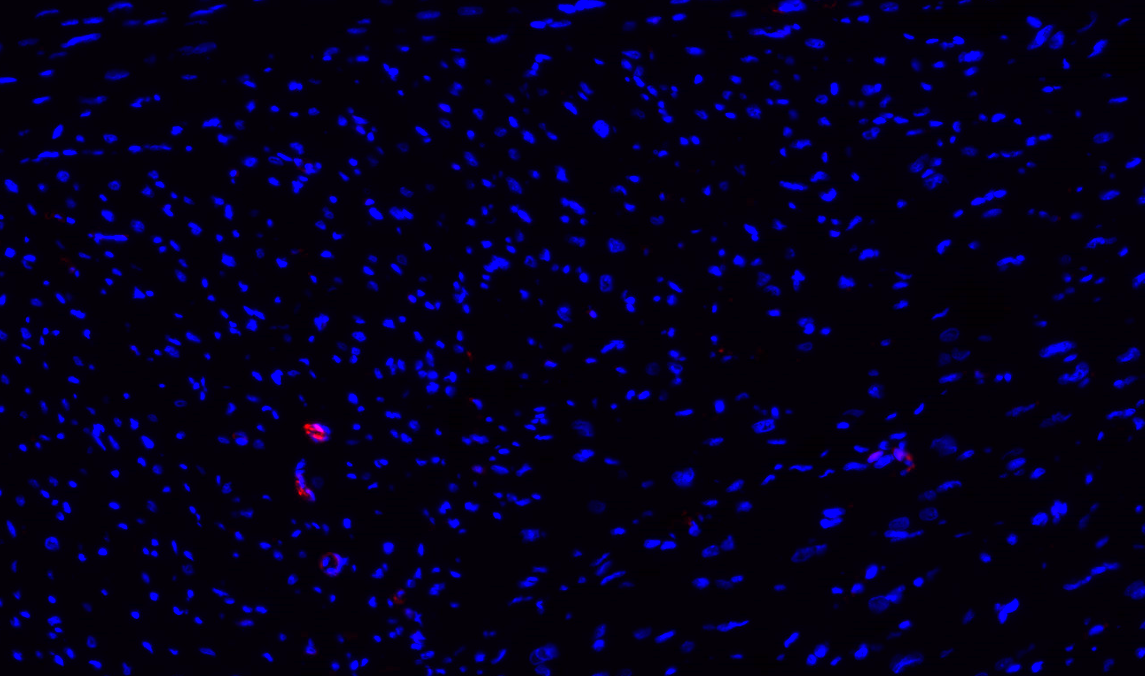

Supplement: S1 Data — (ZIP) [file pone.0295566.s002.zip › minimal underlying data/Fig2/αSMA/sham+vericiguat1_p0.tif]

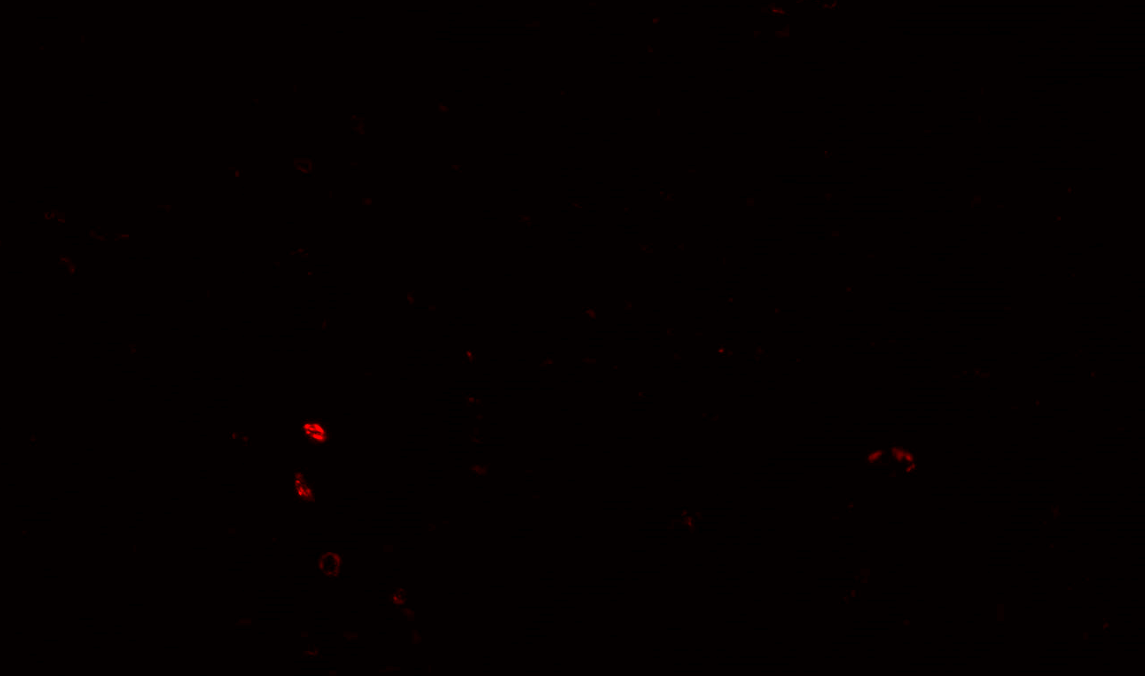

Supplement: S1 Data — (ZIP) [file pone.0295566.s002.zip › minimal underlying data/Fig2/αSMA/sham+vericiguat2_p0.tif]

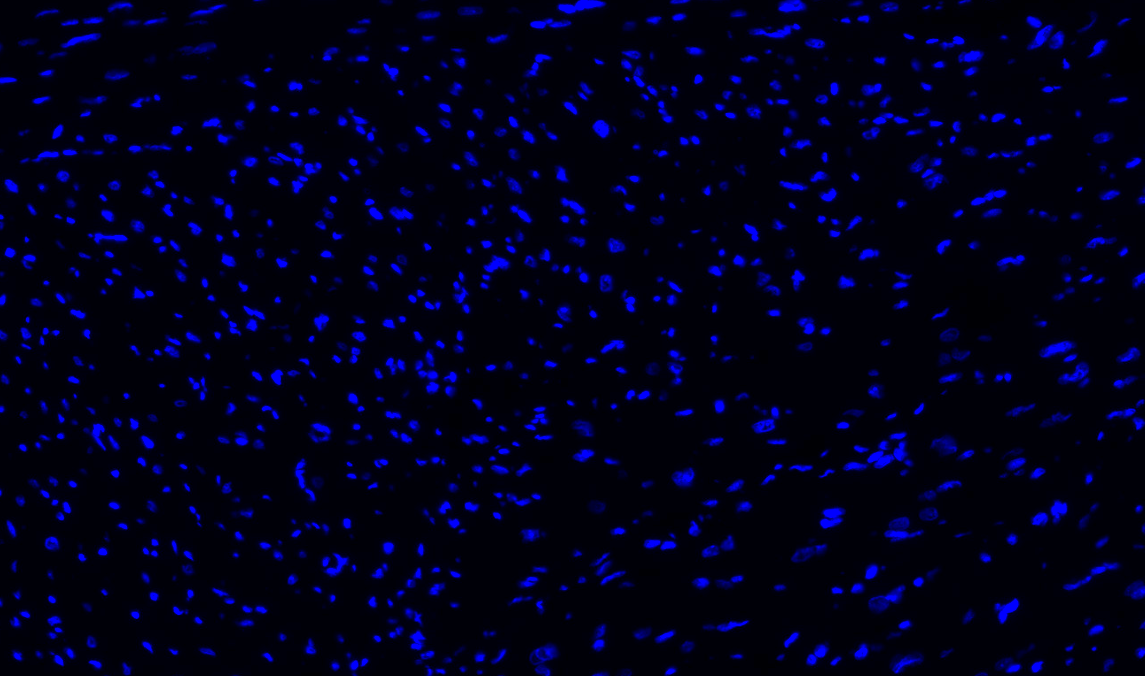

Supplement: S1 Data — (ZIP) [file pone.0295566.s002.zip › minimal underlying data/Fig2/αSMA/sham+vericiguat3_p0.tif]

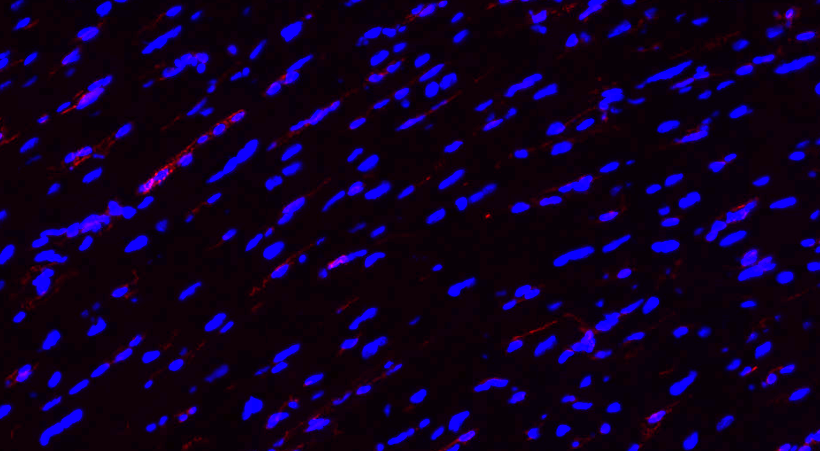

Supplement: S1 Data — (ZIP) [file pone.0295566.s002.zip › minimal underlying data/Fig2/αSMA/sham1_p0.tif]

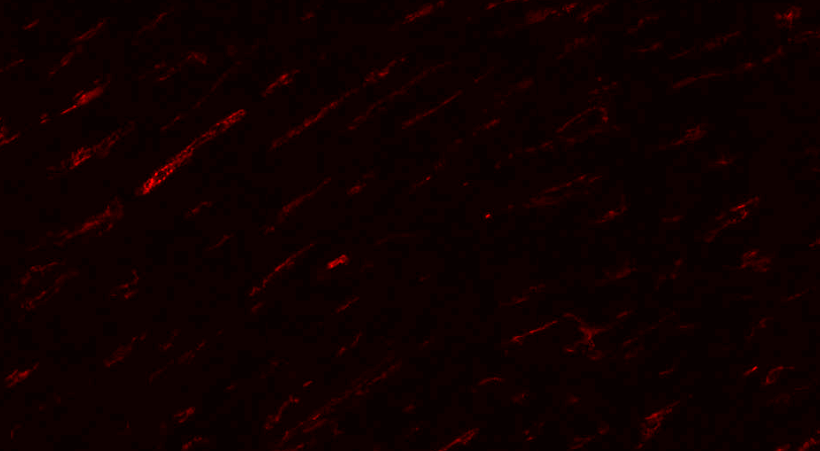

Supplement: S1 Data — (ZIP) [file pone.0295566.s002.zip › minimal underlying data/Fig2/αSMA/sham2_p0.tif]

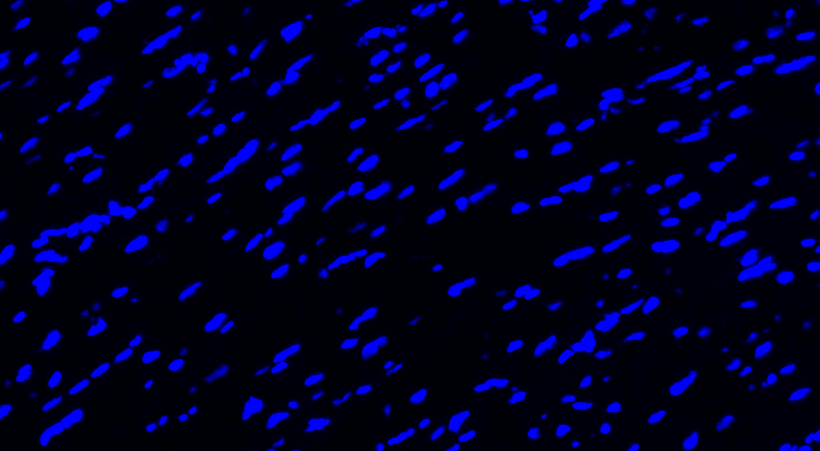

Supplement: S1 Data — (ZIP) [file pone.0295566.s002.zip › minimal underlying data/Fig2/αSMA/sham3_p0.tif]

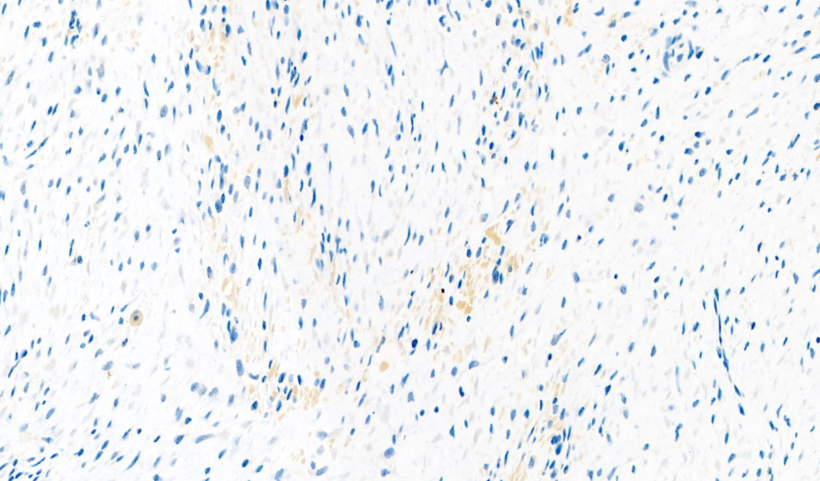

Supplement: S1 Data — (ZIP) [file pone.0295566.s002.zip › minimal underlying data/Fig3/CD16/IR+vericiguat.tif]

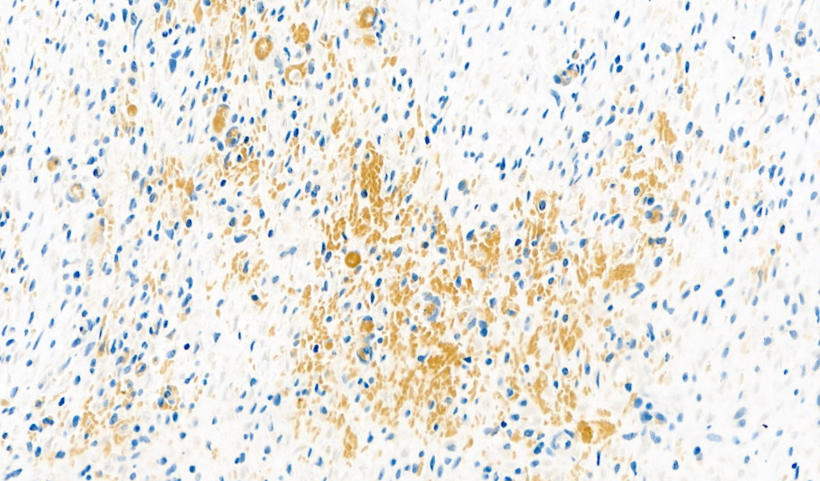

Supplement: S1 Data — (ZIP) [file pone.0295566.s002.zip › minimal underlying data/Fig3/CD16/IR.tif]

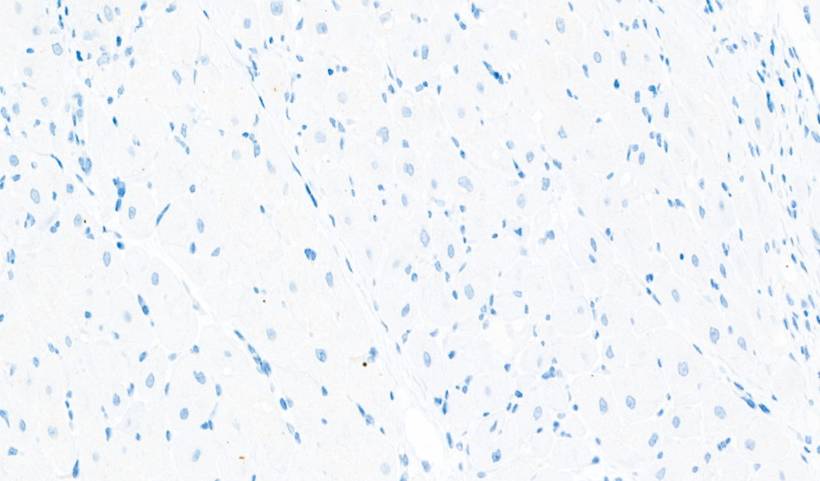

Supplement: S1 Data — (ZIP) [file pone.0295566.s002.zip › minimal underlying data/Fig3/CD16/sham+vericiguat.tif]

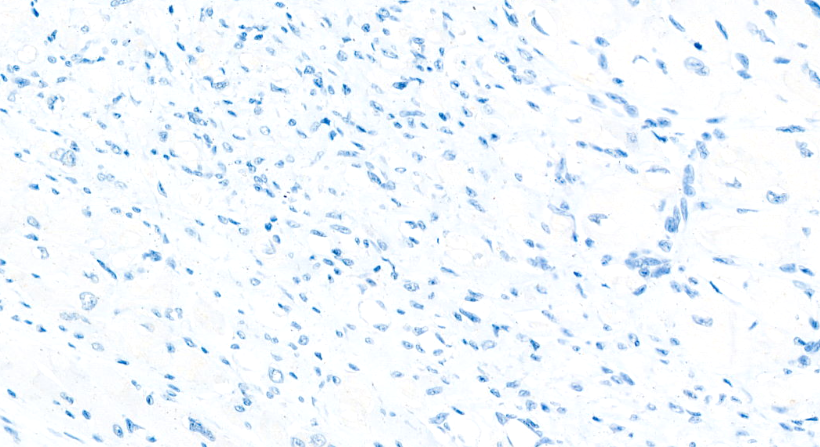

Supplement: S1 Data — (ZIP) [file pone.0295566.s002.zip › minimal underlying data/Fig3/CD16/sham.tif]

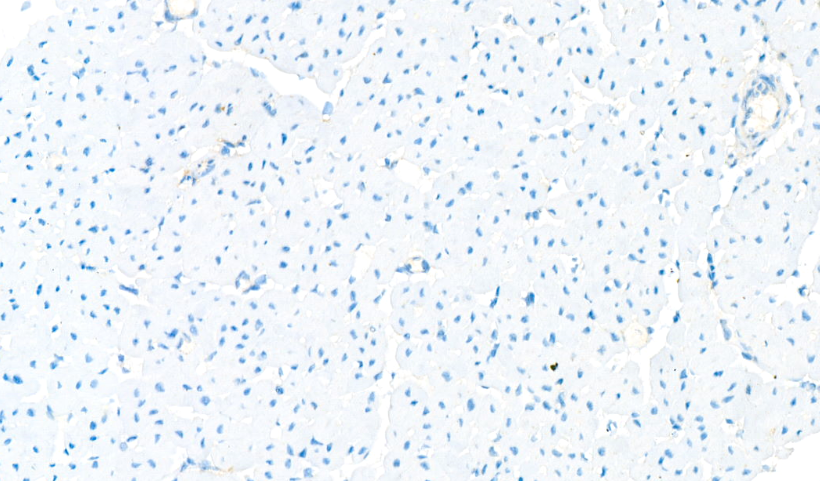

Supplement: S1 Data — (ZIP) [file pone.0295566.s002.zip › minimal underlying data/Fig3/CD68/2sham.tif]

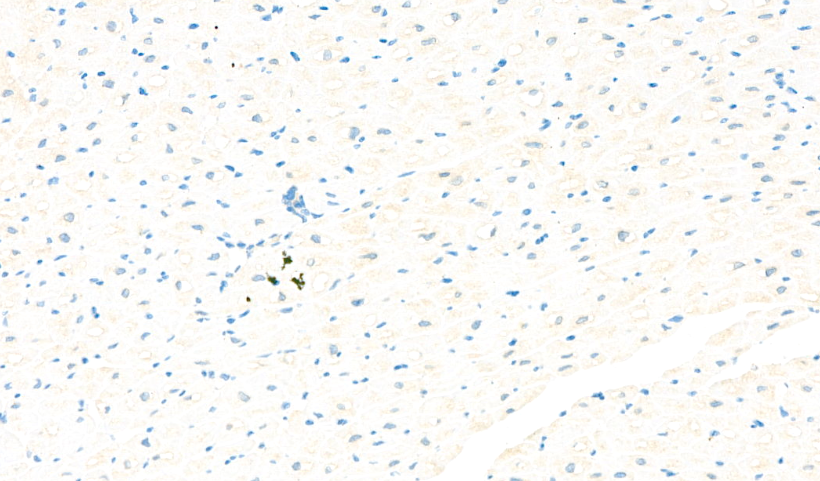

Supplement: S1 Data — (ZIP) [file pone.0295566.s002.zip › minimal underlying data/Fig3/CD68/IR+vericiguat.tif]

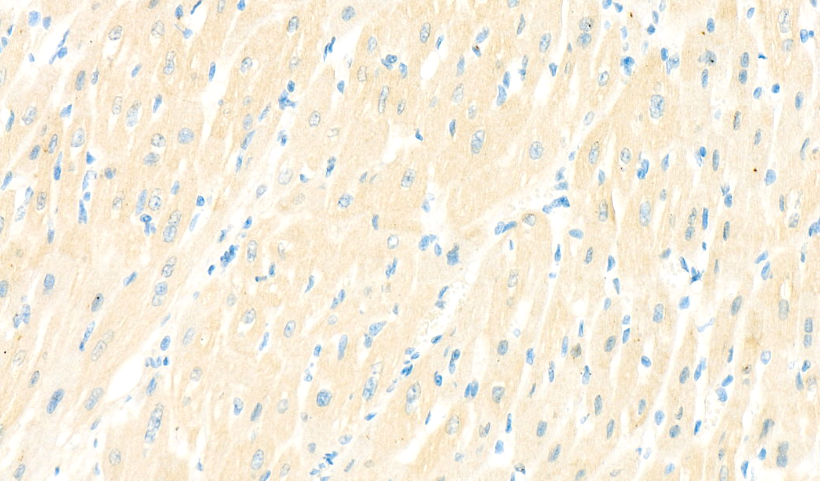

Supplement: S1 Data — (ZIP) [file pone.0295566.s002.zip › minimal underlying data/Fig3/CD68/IR.tif]

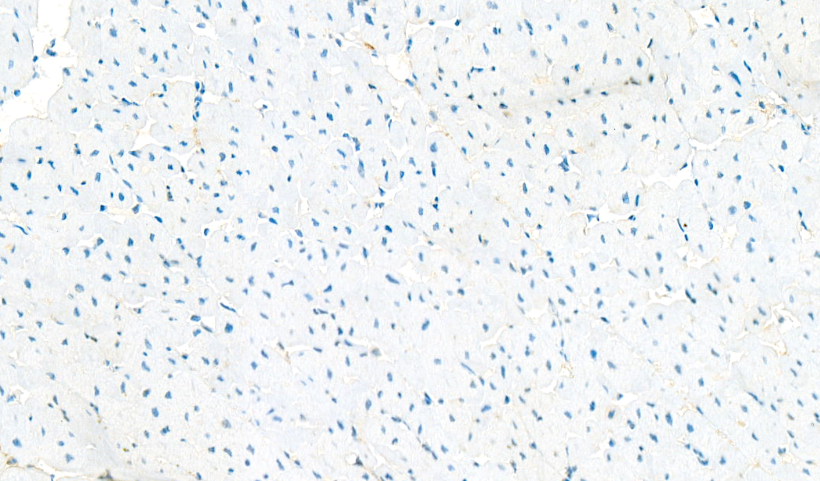

Supplement: S1 Data — (ZIP) [file pone.0295566.s002.zip › minimal underlying data/Fig3/CD68/sham+vericiguat.bmp]

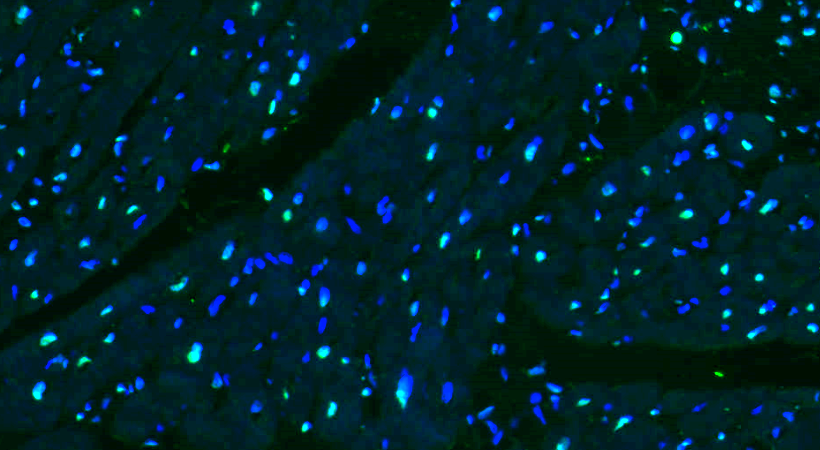

Supplement: S1 Data — (ZIP) [file pone.0295566.s002.zip › minimal underlying data/Fig4/TUNEL/2IR2.tif]

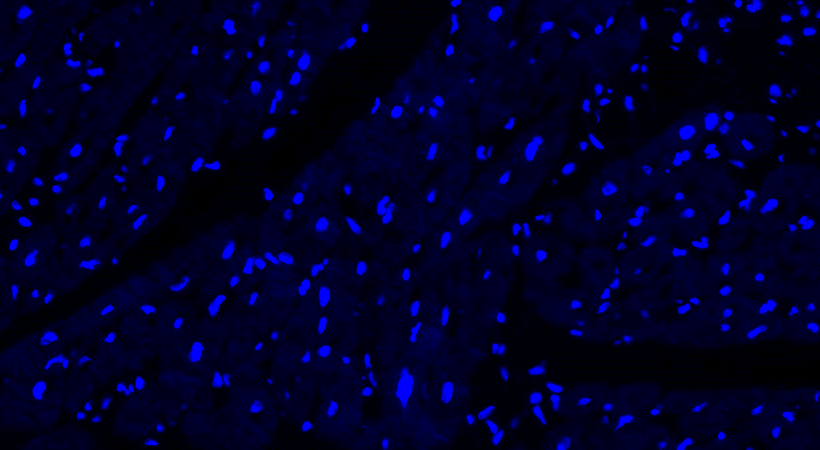

Supplement: S1 Data — (ZIP) [file pone.0295566.s002.zip › minimal underlying data/Fig4/TUNEL/2IR3.tif]

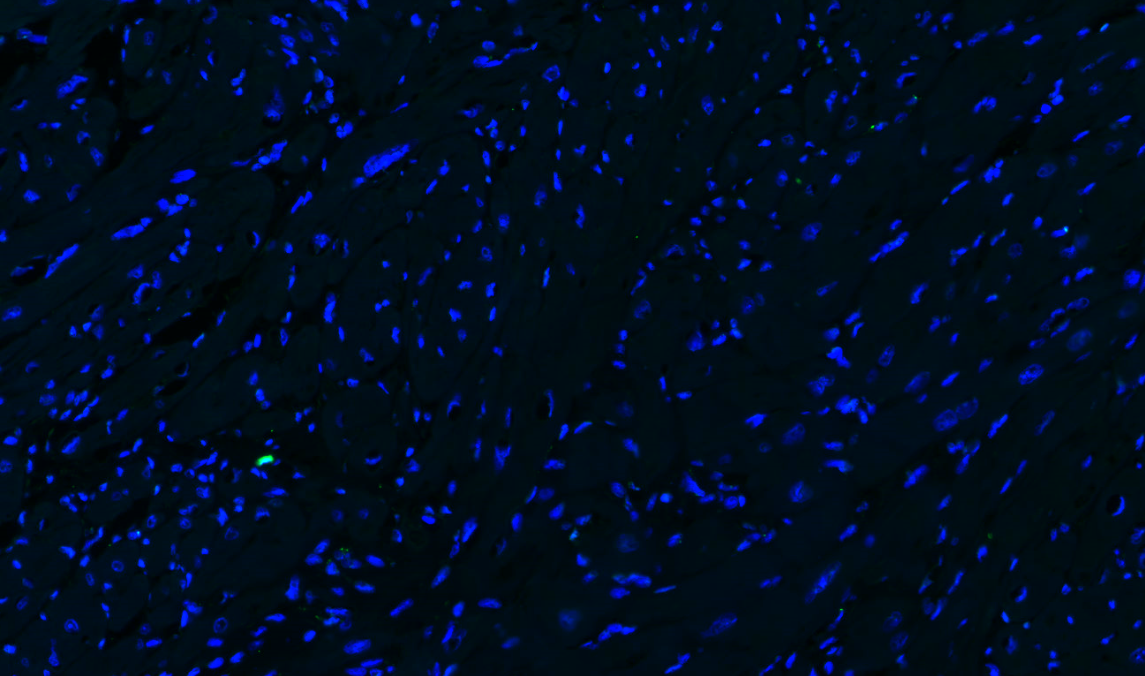

Supplement: S1 Data — (ZIP) [file pone.0295566.s002.zip › minimal underlying data/Fig4/TUNEL/2sham+vericiguat.tif]

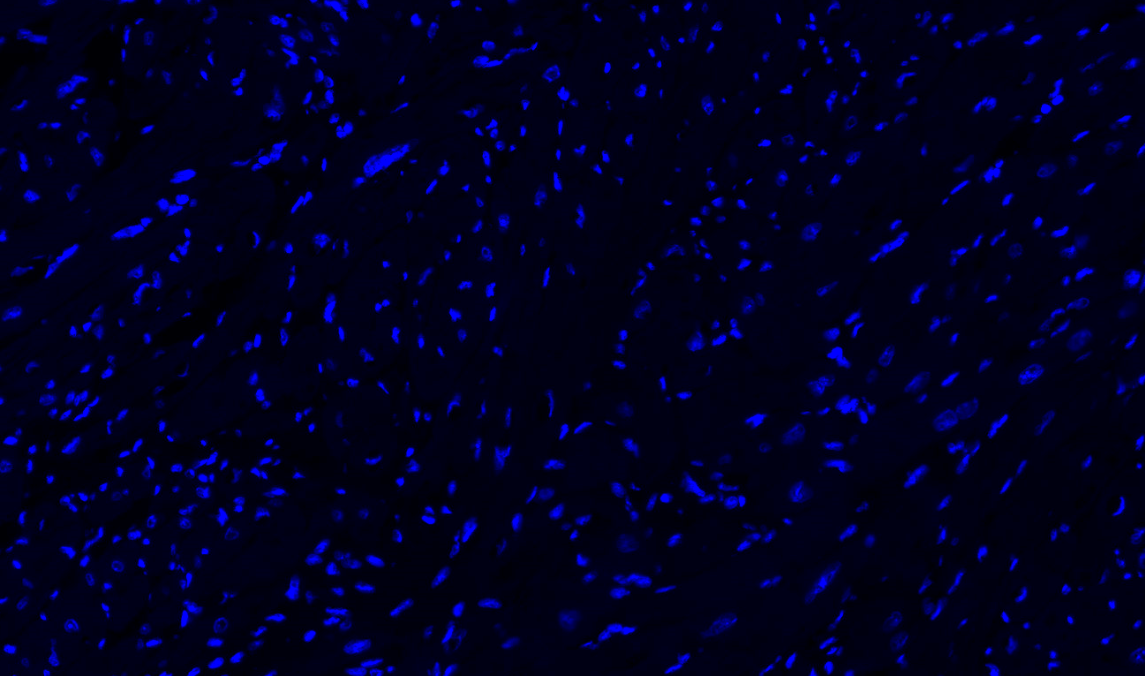

Supplement: S1 Data — (ZIP) [file pone.0295566.s002.zip › minimal underlying data/Fig4/TUNEL/2sham+vericiguat2.tif]

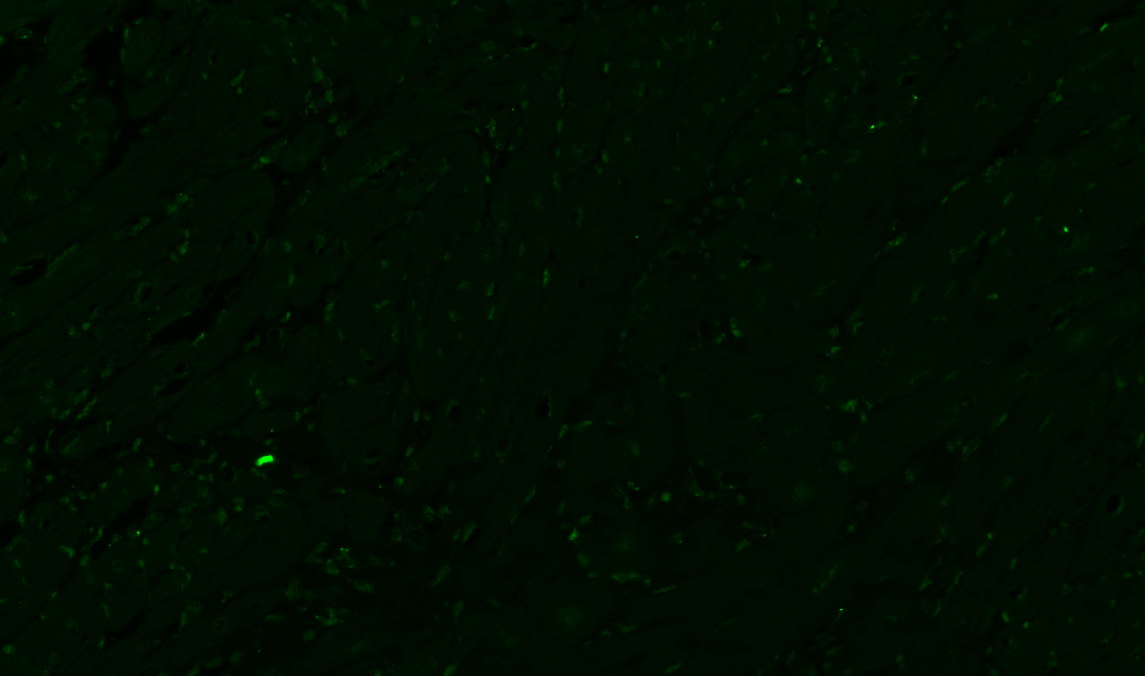

Supplement: S1 Data — (ZIP) [file pone.0295566.s002.zip › minimal underlying data/Fig4/TUNEL/2sham+vericiguat3_p0.tif]

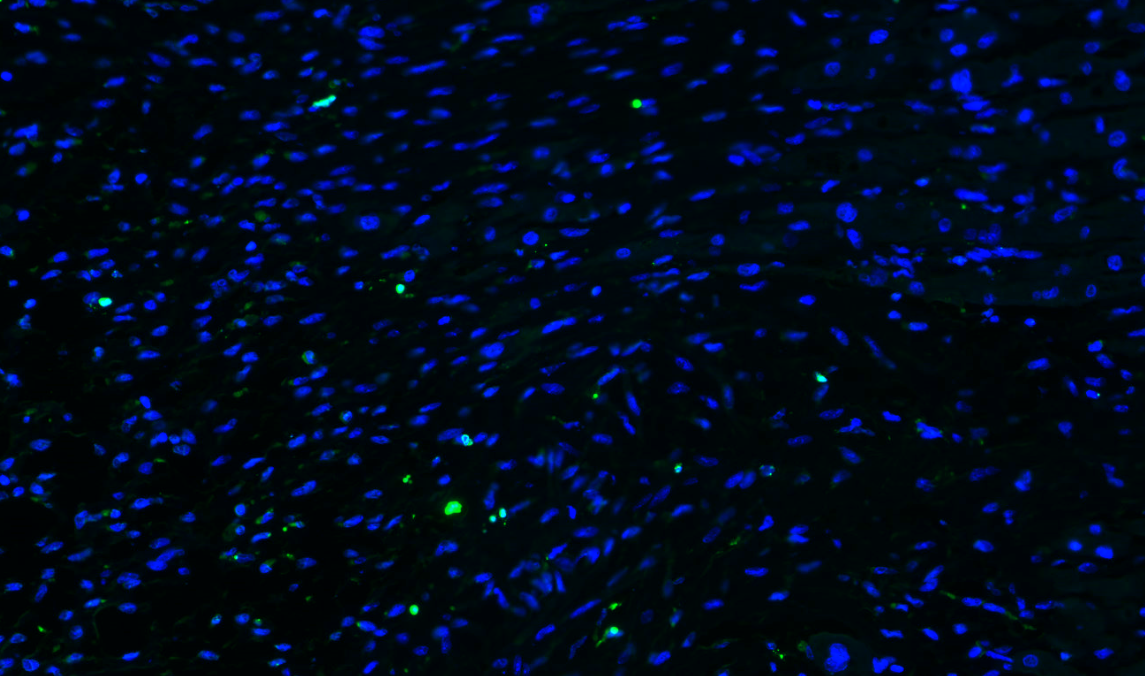

Supplement: S1 Data — (ZIP) [file pone.0295566.s002.zip › minimal underlying data/Fig4/TUNEL/IR+vericiguat.tif]

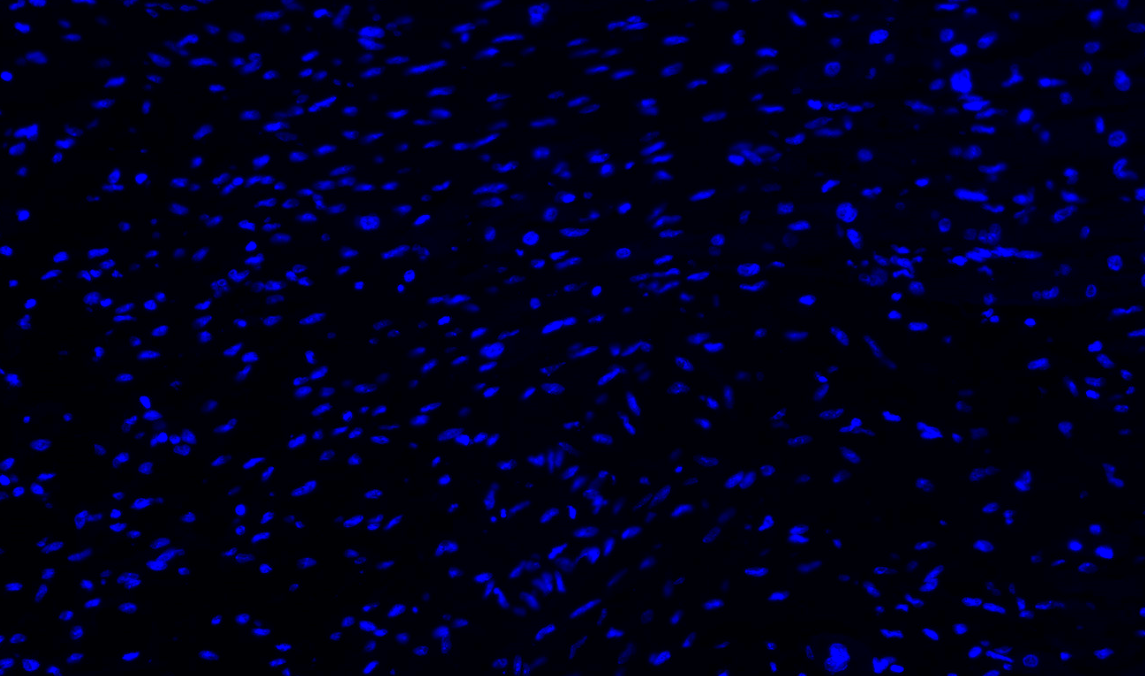

Supplement: S1 Data — (ZIP) [file pone.0295566.s002.zip › minimal underlying data/Fig4/TUNEL/IR+vericiguat2.tif]

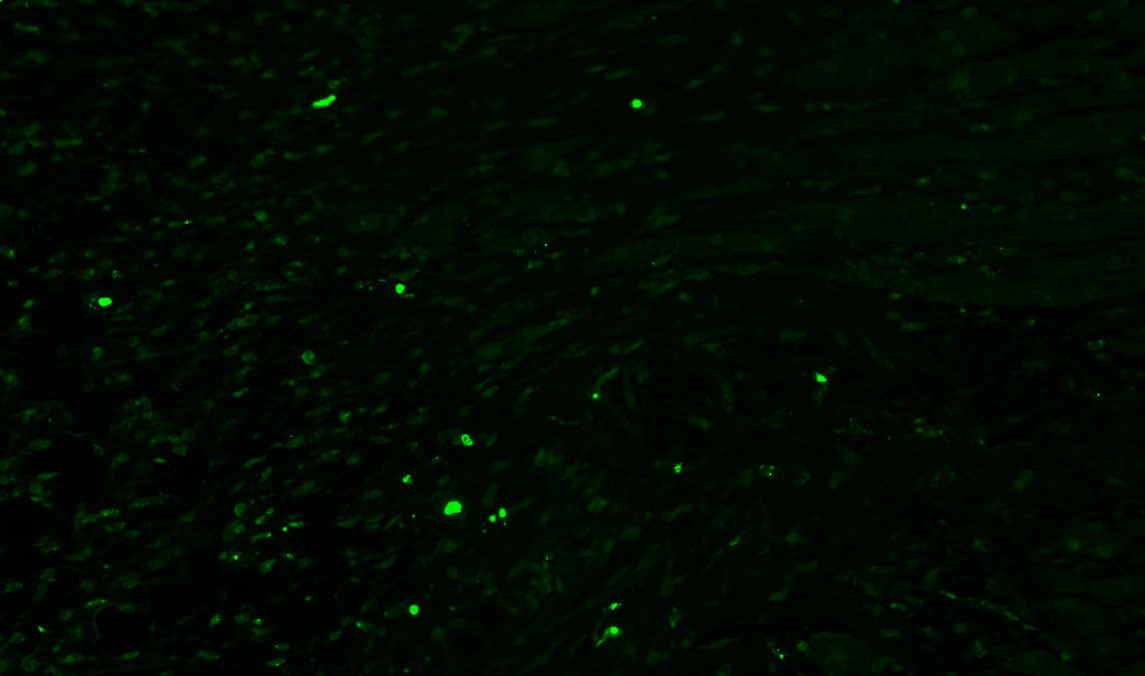

Supplement: S1 Data — (ZIP) [file pone.0295566.s002.zip › minimal underlying data/Fig4/TUNEL/IR+vericiguat3.tif]

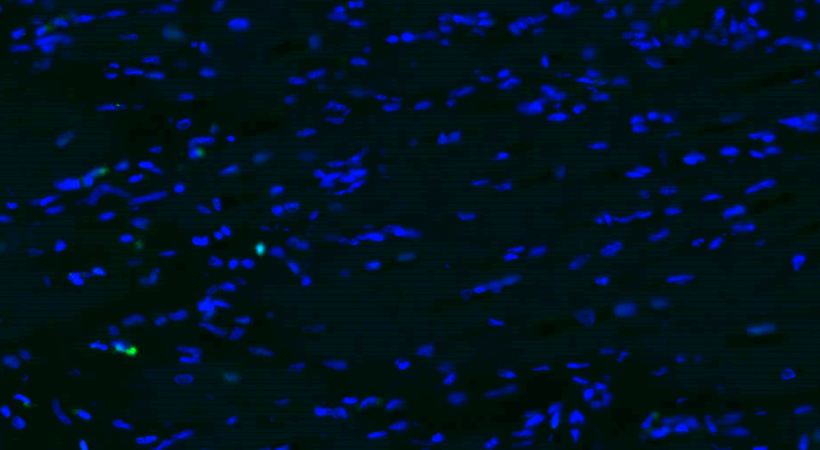

Supplement: S1 Data — (ZIP) [file pone.0295566.s002.zip › minimal underlying data/Fig4/TUNEL/sham.tif]

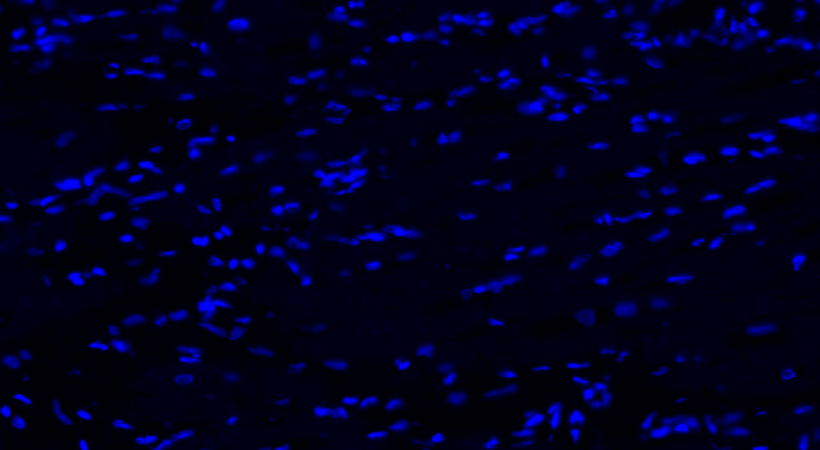

Supplement: S1 Data — (ZIP) [file pone.0295566.s002.zip › minimal underlying data/Fig4/TUNEL/sham1.tif]

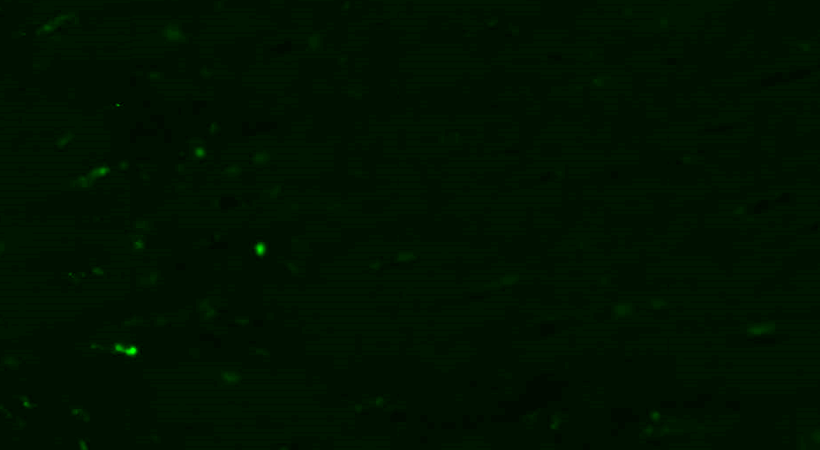

Supplement: S1 Data — (ZIP) [file pone.0295566.s002.zip › minimal underlying data/Fig4/TUNEL/sham2.tif]

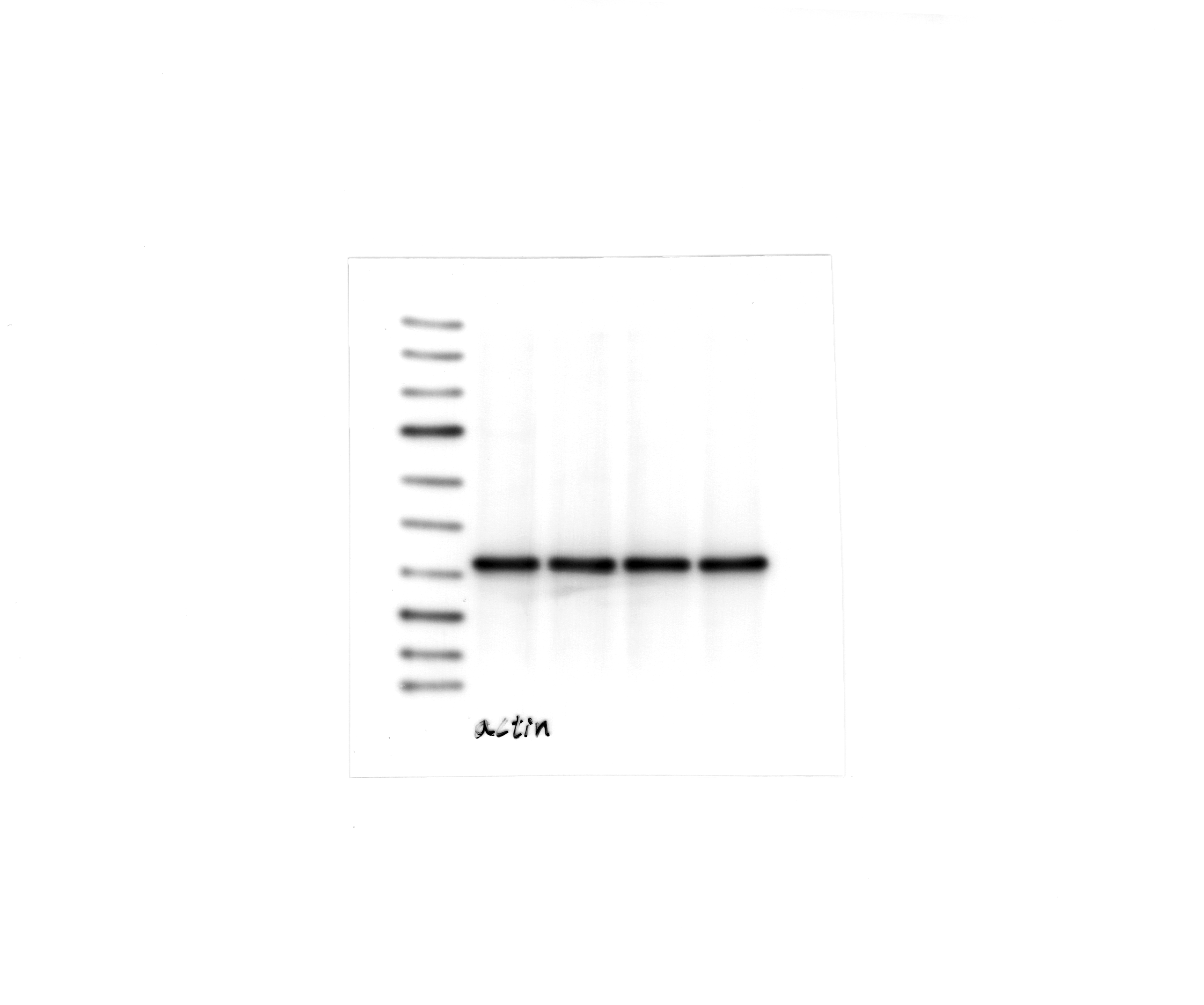

Supplement: S1 Data — (ZIP) [file pone.0295566.s002.zip › minimal underlying data/Fig4/western/actin.tif]

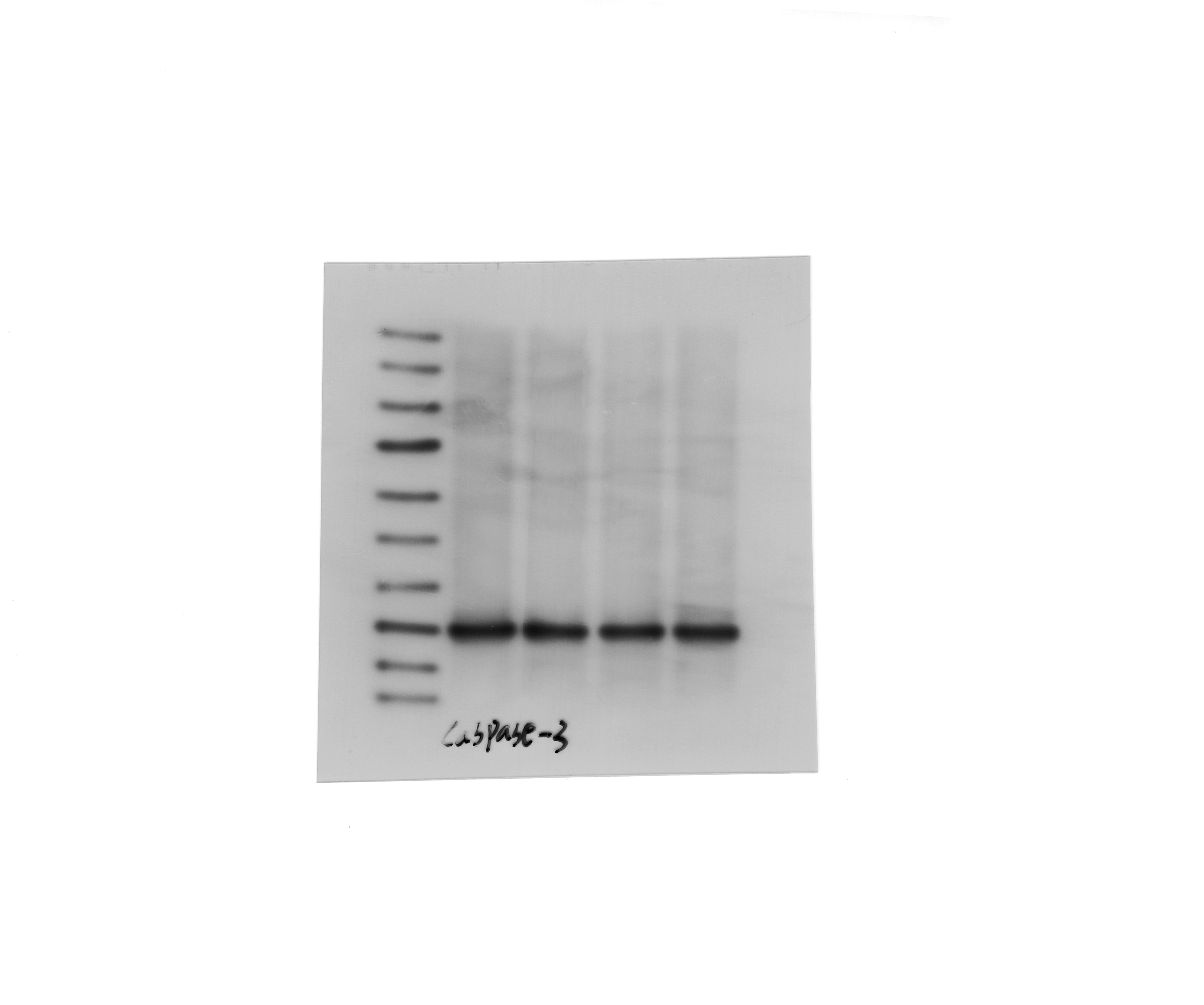

Supplement: S1 Data — (ZIP) [file pone.0295566.s002.zip › minimal underlying data/Fig4/western/caspase-3.tif]

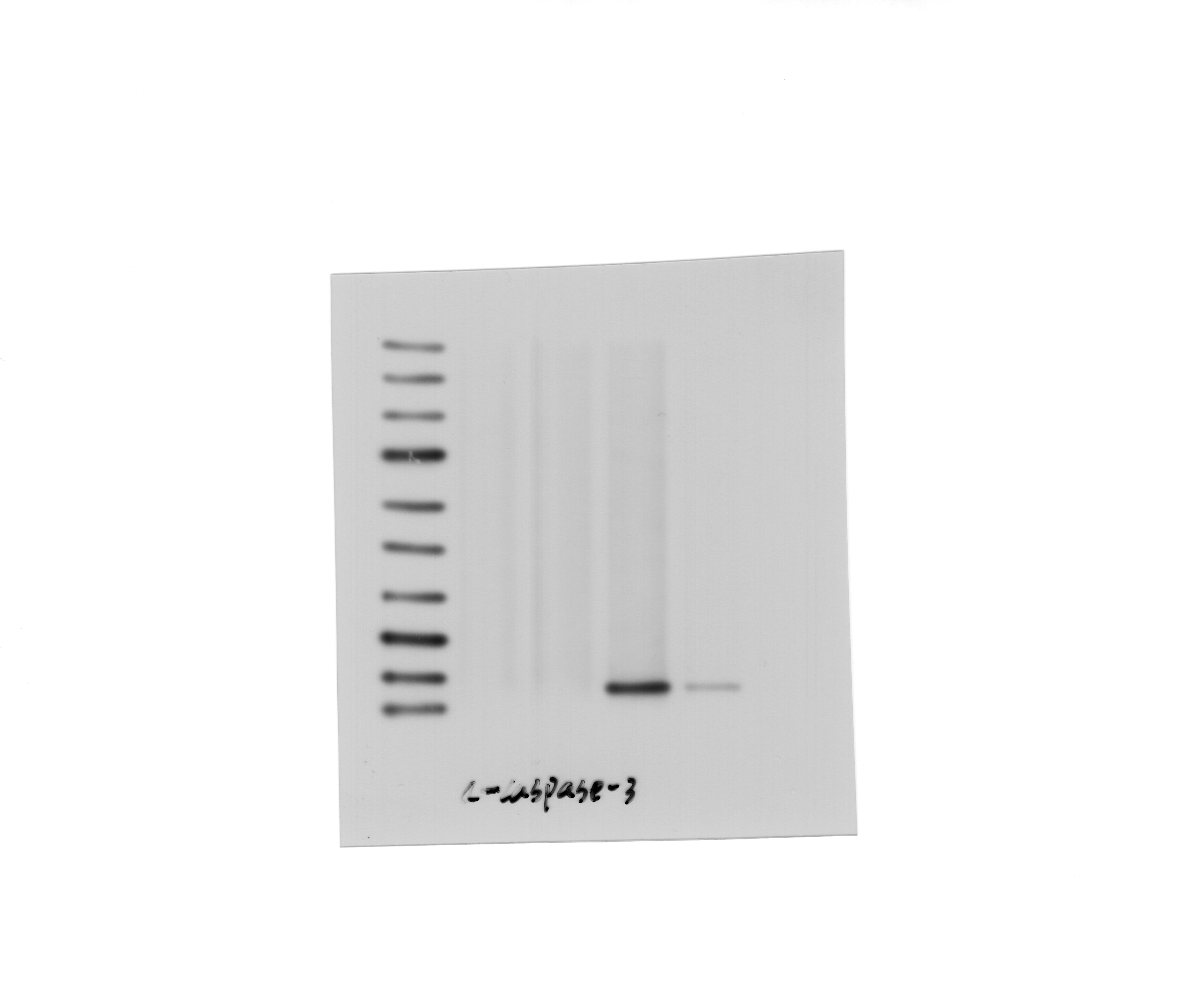

Supplement: S1 Data — (ZIP) [file pone.0295566.s002.zip › minimal underlying data/Fig4/western/cleaved-caspase-3.tif]

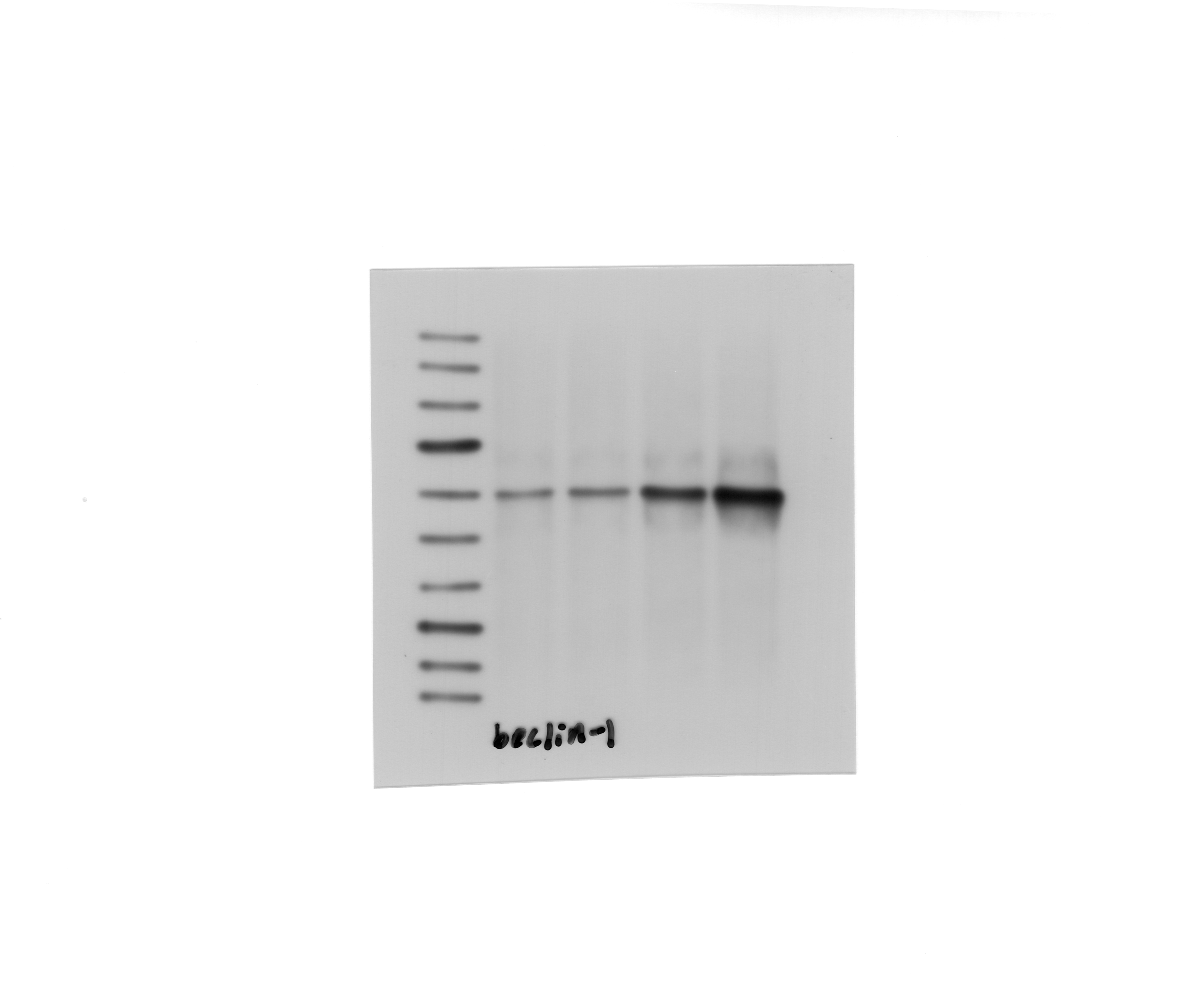

Supplement: S1 Data — (ZIP) [file pone.0295566.s002.zip › minimal underlying data/Fig5/Beclin 1.tif]

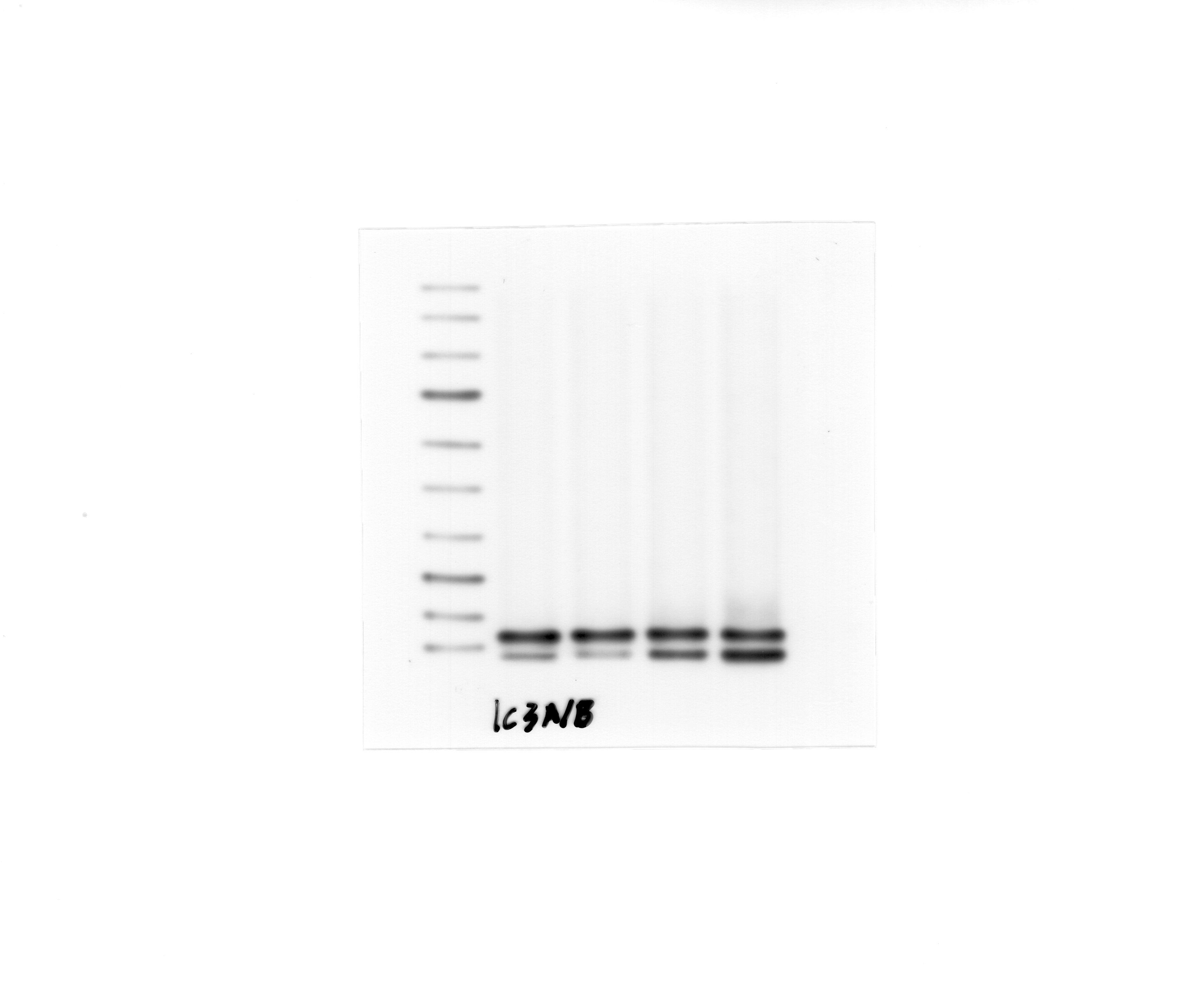

Supplement: S1 Data — (ZIP) [file pone.0295566.s002.zip › minimal underlying data/Fig5/LC3.tif]

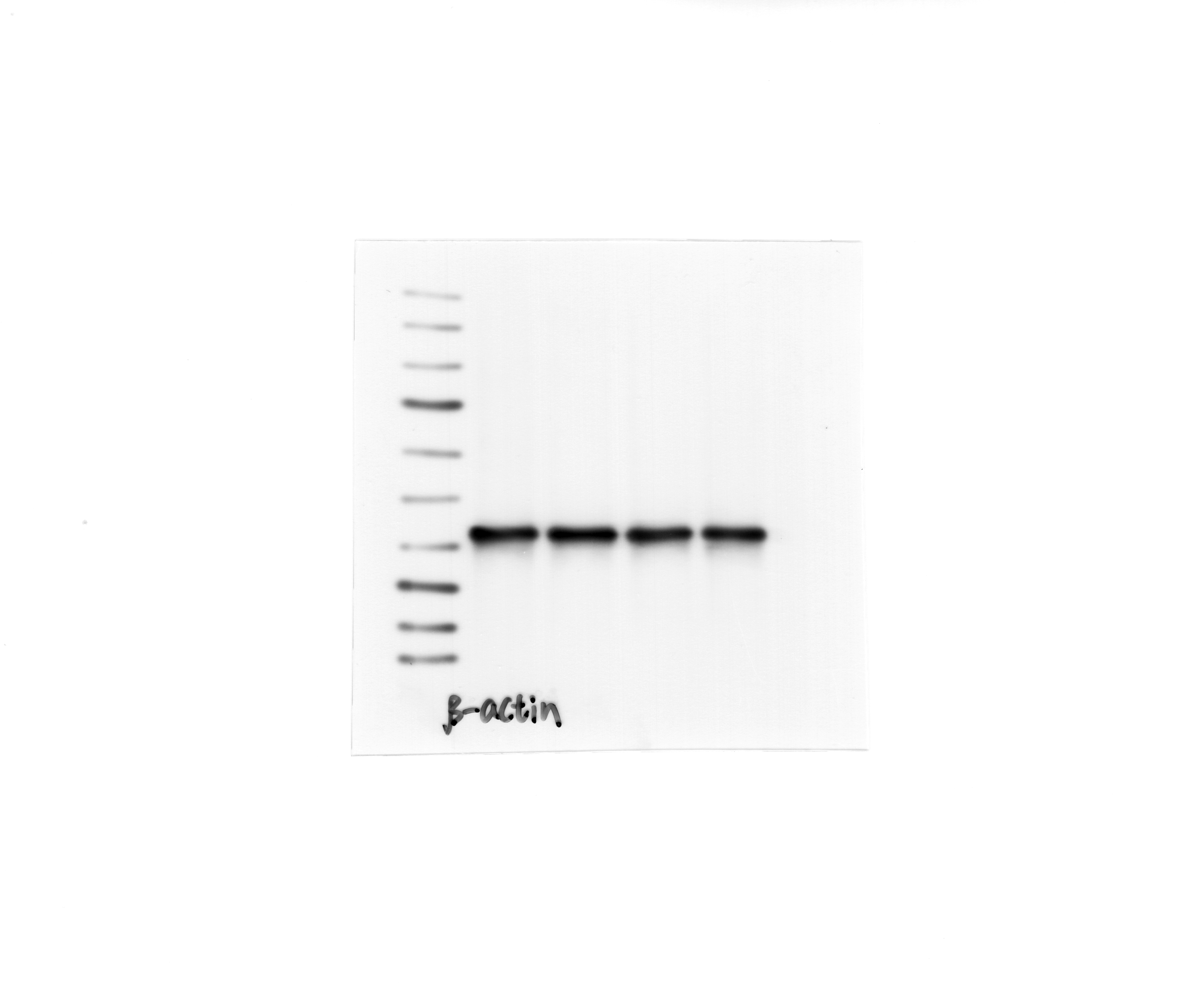

Supplement: S1 Data — (ZIP) [file pone.0295566.s002.zip › minimal underlying data/Fig5/β-actin.tif]
